# Public parks utilization and citizen satisfaction in Bangkok Metropolitan: An integrated theoretical model for tropical urban health

**DOI:** 10.1371/journal.pone.0354172

**Published:** 2026-07-27

**Authors:** Wichian Chanthanet

**Affiliations:** Faculty of Public Administration Liberal Arts, Krirk University, Bangkok, Thailand; Universiti Teknologi Malaysia - Main Campus Skudai: Universiti Teknologi Malaysia, MALAYSIA

## Abstract

**Background:**

Tropical megacities face escalating non-communicable disease burdens requiring innovative urban health solutions. Despite public parks’ critical role as health infrastructure, limited research exists on utilization patterns and satisfaction in tropical urban contexts, where extreme heat and humidity create unique challenges. The equitable distribution of park benefits across socioeconomic groups remains underexplored in Southeast Asian contexts, a gap this study directly addresses.

**Objective:**

This study developed and validated an integrated theoretical model of public park utilization and citizen satisfaction in tropical environments, identifying key determinants and providing evidence-based recommendations for health-promoting urban design, with attention to socioeconomic and gender equity dimensions.

**Methods:**

We conducted a sequential explanatory mixed-methods study combining the Social Ecological Model, Expectation-Disconfirmation Theory, and Place Attachment Theory. Data were collected from 1,200 park users across 30 Bangkok parks using four-stage stratified random sampling, supplemented by 30 in-depth interviews. Analysis included hierarchical regression, thematic analysis, and comprehensive validity assessment.

**Results:**

Participants (58.3% female, mean age 35.9 years) visited parks 3.3 times weekly for 1.8 hours per session. Park quality emerged as the strongest satisfaction predictor (β  =  0.401–0.607, all p  <  0.001), followed by usage patterns and accessibility. The integrated model explained 61.7–68.5% of satisfaction variance, a relatively high figure that partly reflects the common-method effects inherent in self-report designs (see Limitations). All nine hypotheses were confirmed with strong correlations (r  =  0.554–0.796). Climate-specific challenges included inadequate shade coverage (M  =  2.96/5.0) and insufficient evening lighting (M  =  2.98/5.0). Qualitative analysis revealed parks function as urban oases, social ‘third places,’ and community-building spaces, with heat protection identified as the primary accessibility barrier. Significant socioeconomic gradients were observed, with lower-income users reporting systematically lower satisfaction across all domains.

**Conclusions:**

This study provides a comprehensive integrated theoretical model for tropical urban park utilization, indicating that strategic quality improvements are more strongly associated with satisfaction than quantity expansion. Climate-adaptive features—particularly heat protection and extended evening access—represent essential design requirements differing fundamentally from temperate guidelines. The findings indicate that park benefits are not equitably distributed, with implications for Bangkok Metropolitan Administration (BMA) policies including the 15-minute city park initiative.

## Introduction

Non-communicable diseases (NCDs) have emerged as the leading cause of mortality in urban environments, accounting for 74% of global deaths according to the World Health Organization [1,2]. This epidemiological transition represents one of the most significant public health challenges of the 21st century, with cardiovascular diseases, diabetes, cancer, and chronic respiratory diseases now responsible for approximately 41 million deaths annually worldwide [3]. The burden is particularly pronounced in rapidly developing Asian megacities, where urbanization rates exceed 3% annually, creating unprecedented health challenges as lifestyle changes, environmental degradation, and social disruption converge [4,5].

Bangkok, Thailand’s capital and Southeast Asia’s second-largest metropolitan area, with over 10.7 million inhabitants, exemplifies this phenomenon. The city experiences rising rates of cardiovascular disease (affecting 32% of adults), diabetes (prevalence 8.9%), and mental health disorders, paralleling urban expansion [6]. Recent data from the Thailand National Statistical Office reveals that the prevalence of overweight and obesity among Thai adults increased dramatically from 32.8% in 2014 to 41.2% in 2024, despite growing public awareness of physical activity benefits and government-sponsored health promotion campaigns [7].

This paradox between awareness and action highlights the critical importance of environmental determinants of health, particularly the role of built environments in facilitating or constraining health-promoting behaviors [8,9]. Public parks represent a fundamental component of health-promoting urban infrastructure, providing accessible spaces for physical activity, social interaction, stress reduction, and psychological restoration [10,11]. Unlike private fitness facilities or sports clubs that may exclude lower-income populations, public parks offer democratic access to health-promoting environments regardless of socioeconomic status.

However, the democratic promise of public parks is frequently undermined by structural inequalities in access, quality, and design. A substantial body of research documents persistent disparities in park access and quality along socioeconomic lines [52,55,61,65]. In Bangkok, green space is distributed unequally: central districts average only 1.8 m^2^ of green space per capita, while suburban districts reach 5.7 m^2^ per capita. In the present sample, lower-income residents did not travel measurably farther to reach parks (mean travel distance was comparable across income groups, approximately 4.5–4.9 km), but they rated park physical accessibility significantly lower (M = 3.21 vs. 3.47 for higher-income users, p < 0.001) and reported systematically lower satisfaction across all domains, findings explored throughout this manuscript.

The relationship between park characteristics and health outcomes has been extensively documented in temperate Western contexts [12–17], yet remains poorly understood in tropical urban environments. Studies from high-income countries demonstrate associations between park access and increased physical activity [18,19], reduced obesity rates [20], improved mental health [21,22], and enhanced social cohesion [23,24]. However, these findings may not translate directly to tropical megacities characterized by distinct climatic challenges, cultural practices, governance systems, and socioeconomic conditions.

The tropical climate context presents unique challenges for outdoor physical activity and park utilization. Extreme temperatures often exceeding 35°C, high relative humidity (70–85%), intense solar radiation, and monsoon rainfall patterns create temporal and spatial constraints on outdoor activities that differ fundamentally from temperate environments [26,60]. These climatic factors interact with urban heat island effects, air pollution, and inadequate infrastructure to create complex barriers to park utilization. Bangkok’s urban heat island is both well-documented and intensifying, with Marks and Connell [57] demonstrating that poorer inner-city neighborhoods experience disproportionately higher temperatures. Arifwidodo and Chandrasiri [48] found that urban heat stress in Bangkok poses significant health risks for low-income populations. Air quality data from the Pollution Control Department indicate Bangkok’s AQI averaged 87.3 in the first six months of 2024—in the moderate-to-unhealthy range—with PM2.5 and PM10 driven substantially by vehicle emissions and biomass burning [47,56,60].

It is important to acknowledge that prior research has examined park user experience in Bangkok. Chandrasiri and Arifwidodo [51] documented inequality in active public park use at Benjakitti Park, finding that design and amenities served some socioeconomic groups better than others. Arifwidodo and Chandrasiri [68] documented associations between park characteristics and physical activity patterns, and [49] subsequently demonstrated that targeted park improvements meaningfully increase utilization and park-based physical activity. More recently, Arifwidodo, Chandrasiri, and Rueangsom [50] examined park proximity, physical activity, and well-being under Bangkok’s 15-minute parks policy initiative. The present study builds on this prior work by applying a comprehensive multi-dimensional theoretical framework across a larger, more diverse sample of 30 parks, and by explicitly integrating socioeconomic equity as a cross-cutting analytical dimension.

Furthermore, cultural factors specific to Southeast Asian contexts—including collectivistic social orientations, multi-generational family structures, traditional exercise practices, and religious considerations—may influence park usage patterns in ways not captured by Western-derived theoretical models [27,28]. The rapid pace of urbanization in tropical Asia, combined with resource constraints and competing development priorities, also creates unique policy challenges for park development and maintenance [29,30].

This study addresses these substantial research gaps by developing and validating an integrated theoretical model synthesizing three well-established but previously separate theoretical perspectives: the Social Ecological Model [31], Expectation-Disconfirmation Theory [32], and Place Attachment Theory [33]. These frameworks were selected after systematic review of existing theoretical approaches, based on their demonstrated relevance, complementarity, and applicability to the Bangkok context.

The Social Ecological Model (SEM), developed by Bronfenbrenner [31] and elaborated by Sallis et al. [34], provides a multi-level framework for understanding how individual, interpersonal, organizational, community, and policy factors interact to shape health behaviors. In the park context, the SEM explains how personal factors (demographics, health status), social factors (family support, cultural norms), and environmental factors (park quality, accessibility) combine to influence utilization and satisfaction. The SEM was preferred over models such as the Theory of Planned Behavior because its structural and policy-level determinants are better suited to examining how socioeconomic inequalities shape differential access and experience.

Expectation-Disconfirmation Theory (EDT), derived from consumer psychology by Oliver [32,35], explains satisfaction as the result of comparing experiences against prior expectations. EDT provides a theoretically grounded mechanism for explaining why users with identical park access may report markedly different satisfaction levels—a phenomenon especially pronounced across income groups in this study, where lower-income users may develop downwardly adjusted expectations that can mask underlying inequity.

Place Attachment Theory, drawing on Altman and Low [33] and elaborated by Scannell and Gifford [36], examines the emotional bonds people develop with physical environments. Strong place attachment motivates continued usage, stewardship behaviors, and community engagement. The integration of these three frameworks is theoretically coherent: the SEM provides the structural scaffolding (multi-level determinants), EDT provides the psychological mechanism (expectation–experience comparison), and Place Attachment Theory provides the affective and temporal dimension (emotional bonds developing over repeated visits).

**Fig 1:**
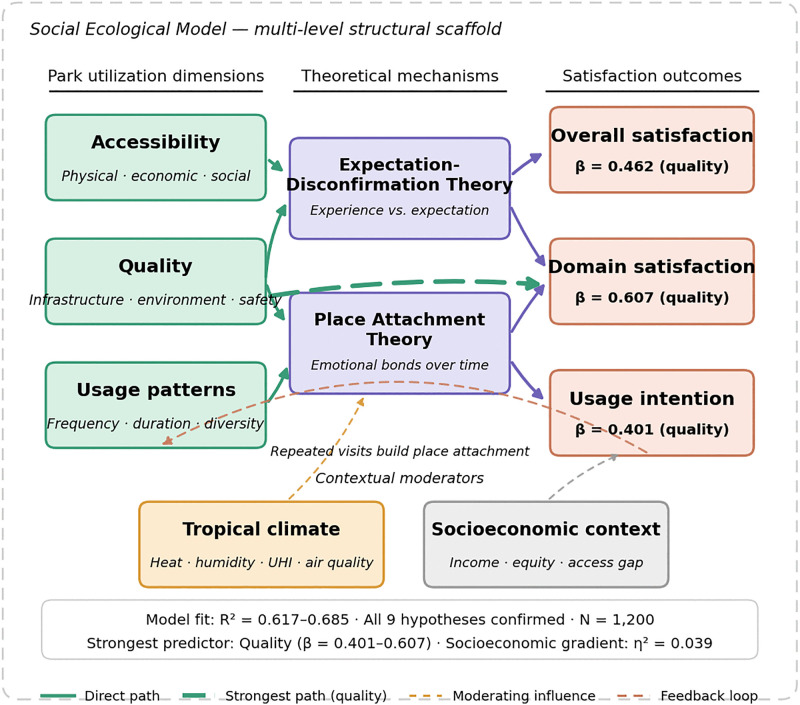
Integrated theoretical model of public park utilization and citizen satisfaction in tropical urban contexts. Teal boxes = park utilization dimensions; purple = theoretical mechanisms; coral = satisfaction outcomes. Arrow thickness reflects relative effect strength. β coefficients from hierarchical regression (Table 4).

A conceptual diagram illustrating the integrated model and hypothesized pathways is presented as Fig 1.

This study aims to: (1) develop and test an integrated theoretical model of public park utilization and citizen satisfaction in tropical urban contexts; (2) identify key determinants of satisfaction specific to tropical climatic and cultural conditions; (3) examine demographic and socioeconomic variations in park experiences, with particular attention to equity; and (4) provide evidence-based recommendations for health-promoting urban design in Southeast Asian cities, with reference to existing BMA policies. Based on the integrated theoretical framework, nine specific hypotheses were formulated (H1–H3: park accessibility, quality, and usage patterns positively correlate with overall satisfaction; H4–H6: with domain-specific satisfaction; H7–H9: with continued usage intention).

## Materials and methods

### Study design and philosophical approach

This study employed a sequential explanatory mixed-methods design [37], in which quantitative findings guide the qualitative phase of data collection and analysis. The philosophical foundation rests on pragmatism [38]. The quantitative phase used a cross-sectional survey design to test hypotheses across a large, representative sample. The qualitative phase employed in-depth interviews to explore meanings, experiences, and contextual factors that quantitative measures cannot capture. The sequential timing allowed qualitative findings to explain and elaborate quantitative results, providing both breadth and depth of understanding.

### Study setting and environmental context

The study was conducted across the Bangkok Metropolitan Area, Thailand. Bangkok’s tropical savanna climate is characterized by three distinct seasons: the hot season (March–May, 29–35°C, humidity 75–85%), the rainy season (June–October, 26–32°C with frequent thunderstorms), and the cool season (November–February, 21–30°C, optimal conditions). Bangkok’s urban heat island effect intensifies these challenges [57], with green space coverage at approximately 3.3 m^2^ per person—well below WHO recommendations of 9 m^2^—and distributed unequally across districts.

### Participants and sampling strategy

Target population: Adults aged 18 years or older who had used public parks in the Bangkok Metropolitan Area within the six months preceding data collection. All participants were required to be BMA residents for at least 12 months (inclusion criterion 4), ensuring park experiences reflected habitual local use rather than tourist visits. Research assistants were trained to verify residential status at recruitment; international tourists or short-term visitors were excluded and not approached.

A four-stage stratified random sampling design ensured representative coverage across Bangkok’s socioeconomic and geographic diversity. Bangkok’s 50 administrative districts were classified into three urban-density zones (central, intermediate, suburban) based on 2023 census and urban-development data. Five districts were randomly selected from each zone (15 total); two parks were randomly chosen from each district (30 parks total); and 40 participants were recruited per park through systematic random sampling across morning, afternoon, and evening time slots on both weekdays and weekends over a four-week period.

Sample size was determined through statistical power analysis using G*Power 3.1.9.7. For hierarchical multiple regression with medium effect size (f^2^ = 0.15), α = 0.05, power = 0.80, and up to 10 predictors, the minimum required sample was 139 participants. The final sample of 1,200 accounts for the design effect from clustered sampling (factor = 2.0), anticipated subgroup analyses, and potential missing data. Qualitative participants (n = 30; 12 female, 18 male; age range 19–67, mean 41.2 years) were selected through purposive maximum variation sampling to capture diversity across age, gender, income, and usage patterns.

### Data collection procedures

Quantitative data collection was conducted from September 23, 2024, to May 31, 2025, spanning three seasons to capture representative usage patterns. A 72-item structured questionnaire was administered through face-to-face interviews by eight trained research assistants. Questionnaire domains included demographics and background (8 items), park utilization dimensions—accessibility (13 items across physical, economic, and social sub-dimensions), quality (15 items across physical infrastructure, environmental quality, and safety/security sub-dimensions), and usage patterns (4 items)—and satisfaction measures—overall satisfaction (3 items), domain-specific satisfaction (15 items across five domains: facilities, safety, cleanliness/maintenance, atmosphere/environment, and access/convenience), and continued usage intention (9 items). All items used 5-point Likert scales and underwent rigorous translation and back-translation procedures.

Semi-structured interviews lasting 45–60 minutes explored participants’ lived experiences, meanings, and suggestions for park improvements. Interviews were conducted in Thai or English by trained bilingual interviewers, audio-recorded with permission, and transcribed verbatim. Field notes captured nonverbal expressions and environmental context.

### Measurement instrument development and validity

A five-expert panel reviewed all items using structured Content Validity Index procedures [39]. Panel members included experts in urban planning, environmental psychology, public health, mixed-methods research, and Thai cultural context. The final instrument achieved excellent content validity (S-CVI/Ave = 0.94, S-CVI/UA = 0.89).

Construct validity was assessed through a split-half validation strategy. Exploratory Factor Analysis (EFA) was conducted on one half of the sample (n = 600) using principal component analysis with promax rotation. A three-factor solution across all 32 park-utilization items explained 54.4% of total variance (KMO = 0.976, Bartlett’s χ²(496) = 10,185.1, p < 0.001; primary loadings 0.59–0.81, no cross-loadings > 0.40), with the three factors corresponding to quality (15 items), accessibility (13 items), and usage patterns (4 items). Confirmatory Factor Analysis (CFA) was conducted on the remaining split-half (n = 600) using AMOS 28.0, yielding acceptable to excellent model fit (χ²/df = 2.14, CFI = 0.952, TLI = 0.948, RMSEA = 0.043 [90% CI: 0.038–0.048], SRMR = 0.051). Internal consistency was excellent (Cronbach’s α = 0.789–0.952). Test-retest reliability (n = 120, 14-day interval) yielded ICC values of 0.76–0.91.

Regarding the use of parametric statistics with Likert-scale data: we adopt the position, consistent with Norman [59] and Sullivan and Artino [62], that parametric tests are robust to Likert-scale data when scales contain five or more response categories, sample sizes are large (n ≥ 200), and data are not severely non-normal. All three conditions were met in the present study. Normality was assessed through Shapiro-Wilk tests (all p > 0.05 for composite scale scores), Q-Q plot inspection, and skewness/kurtosis statistics (all within ±2.0). Non-parametric alternatives (Spearman correlations, Mann-Whitney U, Kruskal-Wallis) were computed for key analyses and produced substantively identical conclusions.

### Qualitative thematic analysis

Thematic analysis followed Braun and Clarke’s [40] reflexive six-phase framework. Phase 1 (Data Familiarization): the author and a trained research assistant independently read all transcripts multiple times and wrote reflective memos. Phase 2 (Initial Coding): systematic line-by-line coding generated 847 codes across 30 transcripts. Phase 3 (Theme Searching): codes were collated through affinity mapping, producing 23 initial candidate themes. Phase 4 (Theme Reviewing): themes were tested against the full data corpus for internal coherence and external heterogeneity, reducing them to nine. Phase 5 (Theme Defining): six final themes were defined with clear boundaries. Phase 6 (Report Writing): exemplary extracts were selected to illustrate each theme. The author and a trained research assistant independently coded 20% of transcripts (Cohen’s κ = 0.83, excellent agreement). Reflexivity was maintained through audit trails and member-checking with five participants. This study was conducted and authored by a single researcher. The coding framework, all analytic decisions, theme definition, interpretation, and writing were carried out by the author; trained research assistants contributed only to data collection and to the independent second-coding of a 20% subsample for reliability assessment, working under the author’s direct supervision and following the author’s coding manual. This supporting role did not meet ICMJE authorship criteria and is recognized in the Acknowledgments.

### Mixed-methods integration

Quantitative and qualitative findings were integrated through three primary strategies consistent with Creswell and Plano Clark’s [41] sequential explanatory design typology. First, data transformation: qualitative themes were quantified through frequency analysis across transcripts, enabling direct comparison with quantitative distributions (e.g., Theme 3 frequency of 73% corroborated the lowest-rated quantitative item, shaded walkways M = 2.96). Second, joint displays: quantitative and qualitative results were presented side-by-side for each thematic domain, systematically examining convergence and divergence. Third, meta-inferences: integrated conclusions were developed by the author, drawing on the coded outputs prepared with research-assistant support, synthesizing both data strands into theoretical propositions that directly informed the policy framework.

### Data analysis strategy

Statistical analysis employed IBM SPSS Statistics 28.0. Procedures included descriptive analysis, Pearson correlation (with Bonferroni correction for multiple comparisons), and hierarchical multiple regression (Step 1: demographic controls; Step 2: park utilization dimensions). Clustered data structure (participants nested within 30 parks) was addressed by including park-level urban-zone stratum as a control variable and verifying homogeneity of regression slopes across parks. Multicollinearity was assessed through VIF values (all VIF < 3.0, well below the threshold of 10). Common method variance was examined using Harman’s single-factor test; the first unrotated factor accounted for 28.3% of total variance (< 50% threshold; Podsakoff et al. [66]), suggesting common method bias is not a substantial concern. One-way ANOVA with Tukey’s HSD post-hoc tests were conducted for demographic comparisons. Because the demographic predictors—particularly education and income—are themselves inter-correlated, collinearity diagnostics were inspected for the full predictor set: variance inflation factors ranged from 1.00 to 2.06 (Table 4), well below conventional thresholds. Inter-correlation among the demographic controls therefore did not destabilize the coefficient estimates, and the small demographic coefficients reported below should be read as adjusted associations net of shared variance rather than as independent effects.

### Ethical considerations

The study received full ethics approval from the Human Research Ethics Committee of Krirk University (Protocol #020300/79, approved 23 September 2024), in accordance with the Declaration of Helsinki and Thai national research ethics guidelines. All participants provided written informed consent. Participants consented to audio-recording, verbatim transcription, and the use of anonymized quotations in publications; consent forms specified that no identifying information would be published. Transcripts were de-identified prior to analysis.

## Results

### Participant characteristics

Research assistants approached 1,476 potential participants across 30 parks; 1,200 agreed to participate (response rate 81.3%); 276 declined or were ineligible. Item-level missing data ranged from 0% to 4.5% (mean 3.1%) and were handled by available-case analysis. Gender distribution: 58.3% female (n = 700), 41.2% male (n = 494), 0.5% unspecified (n = 6). Mean age: 35.9 years (SD = 12.6, range 18–72). Educational attainment (valid n = 1,182): primary school or below 3.4%, lower secondary 7.3%, upper secondary/vocational 22.4%, diploma 16.6%, bachelor’s 40.1%, postgraduate (master’s/doctoral) 10.2%. Monthly household income (valid n = 1,140): ≤ 15,000 THB 15.4%, 15,001–25,000 THB 26.9%, 25,001–35,000 THB 24.9%, 35,001–50,000 THB 20.2%, 50,001–75,000 THB 9.4%, > 75,000 THB 3.2%. All participants were confirmed BMA residents of at least 12 months.

### Park usage patterns

Mean visit frequency: 3.3 times/week (SD = 2.0). Mean session duration: 1.8 hours (SD = 0.9). Mean travel distance from home: 4.8 km (SD = 3.0), substantially exceeding the WHO-recommended 400–800 m service radius. Primary transportation modes: private vehicle 42.3%, BTS/MRT 28.5%, bus 19.0%, other 10.1%.

Primary activities (multiple responses allowed): walking/strolling 67.5%, jogging/running 45.2%, sitting/relaxing 38.9%, social activities 35.0%, children’s play 25.0%, and sports/exercise 18.3% (mean 2.3 activities per respondent). Wedding and graduation photography was observed by research assistants at 6 of 30 parks, reflecting parks’ social function beyond recreation.

### Park utilization dimensions

Table 1 presents descriptive statistics for park utilization dimensions. Social accessibility (M = 3.54) was measured across cultural accommodation (degree to which facilities and design are perceived as welcoming to diverse cultural groups, M = 3.61) and safety for diverse users (perceived security for women, elderly, and other groups who may feel vulnerable, M = 3.48). The moderate rating for safety for diverse users aligns with qualitative Theme 4, in which women consistently reported reluctance to use parks after dark due to inadequate lighting.

**Table 1:** Descriptive statistics for park utilization dimensions.

Dimension/Subdimension	M	SD	Min	Max	α
**Accessibility (Overall)**	**3.57**	**0.58**	**1.60**	**5.00**	**0.941**
Physical accessibility	3.36	0.80	1.00	5.00	0.865
Distance to park	3.56	0.93	1	5	—
Pedestrian safety	3.20	0.98	1	5	—
Shaded walkways	2.96	1.03	1	5	—
Transit connection	3.64	0.91	1	5	—
Parking	3.42	1.07	1	5	—
Economic accessibility	3.74	0.72	1.50	5.00	0.827
Travel cost	3.86	0.84	1	5	—
Free admission	4.18	0.73	2	5	—
Opportunity/time cost	3.47	0.97	1	5	—
Travel options	3.45	0.96	1	5	—
Social accessibility	3.53	0.75	1.00	5.00	0.823
Safety for diverse users	3.40	0.94	1	5	—
Opening hours	3.77	0.83	1	5	—
Information access	3.26	1.03	1	5	—
Suitable for all ages	3.68	0.90	1	5	—
**Quality (Overall)**	**3.37**	**0.69**	**1.00**	**5.00**	**0.949**
Physical infrastructure	3.33	0.82	1.00	5.00	0.862
Benches/seating	3.46	1.03	1	5	—
Playground	3.10	1.11	1	5	—
Exercise equipment	3.25	1.08	1	5	—
Walking/cycling paths	3.56	0.93	1	5	—
Landscape	3.29	0.95	1	5	—
Environmental quality	3.45	0.78	1.00	5.00	0.862
Air quality	3.65	0.88	1	5	—
Shade area	3.19	1.06	1	5	—
Ventilation	3.53	0.94	1	5	—
Biodiversity	3.37	1.01	1	5	—
Waste management	3.50	0.96	1	5	—
Safety and security	3.25	0.81	1.00	5.00	0.861
Nighttime lighting	2.98	1.10	1	5	—
Access control	3.35	0.97	1	5	—
Visibility	3.42	0.96	1	5	—
Security presence	3.14	1.06	1	5	—
Staff care	3.38	0.93	1	5	—
**Usage Patterns (Overall)**	**3.48**	**0.79**	**1.22**	**5.00**	**—**

*Note: M = mean; SD = standard deviation; α = Cronbach’s alpha.*

Shaded walkways received the lowest rating of any single item (M = 2.96), consistent with a mismatch between Bangkok’s tropical climate and current park infrastructure. Nighttime lighting inadequacy (M = 2.98) limits evening usage and generates safety concerns disproportionately affecting women. Free admission scored highest (M = 4.18). Lower-income users (≤25,000 THB/month) rated physical accessibility significantly lower than higher-income users (M = 3.21 vs. 3.47, p < 0.001), reflecting greater public-transport dependence and lower perceived accessibility.

### Satisfaction assessment

Table 2 presents satisfaction statistics. Overall satisfaction was moderately high (M = 3.65), with environment satisfaction highest among domain-specific measures (M = 3.67) and safety satisfaction lowest (M = 3.36), consistent with lighting and security concerns. Continued usage intention was strong (M = 3.68).

**Table 2:** Satisfaction assessment across multiple dimensions.

Satisfaction Dimension	M	SD	Min	Max	α
**Overall Satisfaction**	**3.65**	**0.67**	**1.39**	**5.00**	**0.788**
General satisfaction	3.65	0.83	1	5	—
Overall experience	3.58	0.87	1	5	—
Enjoyment	3.70	0.81	2	5	—
**Domain-Specific Satisfaction**	**3.49**	**0.63**	**1.57**	**5.00**	**0.949**
Facilities	3.40	0.85	1.00	5.00	0.781
Safety	3.36	0.84	1.00	5.00	0.800
Cleanliness & maintenance	3.48	0.78	1.00	5.00	0.796
Atmosphere & environment	3.67	0.70	1.33	5.00	0.776
Access & travel convenience	3.45	0.80	1.00	5.00	0.792
**Continued Usage Intention**	**3.68**	**0.70**	**1.21**	**5.00**	**0.911**
Repeat use	3.77	0.71	1.33	5.00	0.776
Recommendation	3.71	0.73	1.00	5.00	0.779
Loyalty/attachment	3.56	0.78	1.00	5.00	0.792

*Note: M = mean; SD = standard deviation; α = Cronbach’s alpha.*

### Hypothesis testing and correlation analysis

All nine study hypotheses were confirmed (Table 3). Quality showed the strongest correlations with all outcomes (r = 0.687–0.796), followed by accessibility (r = 0.624–0.692) and usage patterns (r = 0.554–0.644). All correlations were statistically significant after Bonferroni correction.

**Table 3:** Comprehensive correlation matrix.

Variable	1	2	3	4	5	6
1. Accessibility	—					
2. Quality	0.649	—				
3. Usage Patterns	0.536	0.492	—			
4. Overall Satisfaction	0.692	0.752	0.624	—		
5. Domain Satisfaction	0.647	0.796	0.554	0.834	—	
6. Usage Intention	0.624	0.687	0.644	0.763	0.721	—

Note: **p < 0.001 (two-tailed). N = 1,200. All correlations significant after Bonferroni correction.

**Table 4:** Hierarchical multiple regression results.

Predictors	Overall Satisfaction β	Domain Satisfaction β	Usage Intention β
**Step 1: Control Variables**			
Gender (female = 1)	−0.021 ns	−0.014 ns	0.020 ns
Age (continuous)	−0.002 ns	0.018 ns	0.006 ns
Education level	0.017 ns	0.046*	−0.003 ns
Income level	0.023 ns	0.023 ns	0.037 ns
R²	0.022	0.027	0.023
**Step 2: Park Dimensions**			
Accessibility	0.243**	0.154**	0.156**
Quality	0.462**	0.607**	0.401**
Usage Patterns	0.262**	0.171**	0.363**
R²	0.682	0.685	0.617
ΔR²	0.660	0.658	0.594

Note: *p < 0.05. **p < 0.001. β = standardized regression coefficients. VIF values ranged from 1.00 to 2.06 (all < 3.0), indicating no problematic multicollinearity.

### Hierarchical multiple regression analysis

Three hierarchical multiple regression models examined the relative contribution of park utilization dimensions, controlling for demographics (Step 1) and adding park dimensions (Step 2). Regression equations (standardized coefficients): Overall Satisfaction = −0.021(Gender) − 0.002(Age) + 0.017(Education) + 0.023(Income) + 0.243(Accessibility) + 0.462(Quality) + 0.262(Usage Patterns) + ε; R² = 0.682. Domain-Specific Satisfaction = −0.014(Gender) + 0.018(Age) + 0.046(Education) + 0.023(Income) + 0.154(Accessibility) + 0.607(Quality) + 0.171(Usage Patterns) + ε; R^2^ = 0.685. Continued Usage Intention = 0.020(Gender) + 0.006(Age) − 0.003(Education) + 0.037(Income) + 0.156(Accessibility) + 0.401(Quality) + 0.363(Usage Patterns) + ε; R^2^ = 0.617. The demographic controls were not statistically significant (except education in the domain-satisfaction model, p = 0.007). Table 4 presents full regression results.

Supplementary sensitivity analysis. Because Usage Patterns (a predictor) and Continued Usage Intention (an outcome) were moderately correlated (r = 0.644), the Usage Intention model was re-estimated with Usage Patterns removed to confirm that the result was not an artifact of construct overlap. The three-predictor model (without demographic controls) yielded standardized coefficients of 0.18, 0.40, and 0.35 for accessibility, quality, and usage patterns, respectively (R^2^ = 0.61). Removing Usage Patterns and regressing Continued Usage Intention on accessibility and quality alone yielded standardized coefficients of 0.31 and 0.49, respectively, with R^2^ = 0.53—a reduction of roughly 0.08 in explained variance. Quality therefore remained the dominant modifiable predictor, its standardized weight increasing once the overlapping predictor was removed, and the central conclusion of the study was unchanged. The findings are thus robust to the predictor–outcome overlap discussed in the Limitations. Full results are reported in S1 Table.

### Demographic and socioeconomic variations

Table 5 presents satisfaction differences across demographic categories. Gender and age differences were small and not statistically significant (gender Cohen’s d = 0.04–0.09, all p > 0.10; age η^2^ = 0.002–0.005, with only the domain-satisfaction trend approaching significance, p = 0.051). A significant socioeconomic gradient was observed: high-income participants reported overall satisfaction 0.31 points higher than low-income participants (3.87 vs. 3.56; F = 13.30, p < 0.001, η^2^ = 0.022), with parallel gradients for domain satisfaction and usage intention. Although these effect sizes are modest, the gradient is consistent across all outcomes and reflects a structural inequity in park experience.

**Table 5:** Satisfaction differences across demographic categories.

Category	Overall Satisfaction M(SD)	Domain Satisfaction M(SD)	Usage Intention M(SD)
**Gender**			
Male (n = 494)	3.63 (0.69)	3.46 (0.66)	3.64 (0.71)
Female (n = 700)	3.66 (0.66)	3.51 (0.60)	3.71 (0.70)
Cohen’s d	0.04	0.07	0.09
**Age Groups**			
18–30 years (n = 466)	3.61 (0.66)	3.44 (0.63)	3.65 (0.72)
31–50 years (n = 554)	3.66 (0.67)	3.51 (0.63)	3.69 (0.70)
51 + years (n = 180)	3.70 (0.68)	3.56 (0.60)	3.73 (0.66)
F	1.49 ns	2.98 ns	0.96 ns
η²	0.002	0.005	0.002
**Income Level**			
Low (≤25,000 THB, n = 510)	3.56 (0.68)	3.40 (0.63)	3.57 (0.70)
Medium (25,001–50,000, n = 544)	3.67 (0.67)	3.52 (0.63)	3.73 (0.70)
High (>50,000 THB, n = 146)	3.87 (0.60)	3.68 (0.57)	3.89 (0.65)
F	13.30**	12.31**	15.40**
η²	0.022	0.020	0.025

Note. ** p < 0.001. Values are M (SD). Gender and age differences were not statistically significant; only income showed a significant gradient across all three outcomes (one-way ANOVA with Tukey’s HSD).

### Qualitative thematic analysis

Thematic analysis of 30 in-depth interviews (45–60 minutes each) revealed six major themes. Themes are interconnected: Parks as Urban Oases (Theme 1) enables Social Connection (Theme 2), which deepens Place Attachment (Theme 6). Climate Barriers (Theme 3) and Safety/Maintenance Concerns (Theme 4) represent the primary obstacles to this positive cycle. Technology Integration (Theme 5) represents an emerging dimension intersecting with equity concerns.

#### Theme 1: Parks as urban oases and psychological restoration spaces (80%).

Participants consistently conceptualized parks as essential refuges providing psychological and physiological restoration unavailable elsewhere in the city. This theme aligns with Attention Restoration Theory [43] and was expressed with greatest intensity by lower-income participants, reflecting fewer alternative restorative spaces.

*“The park is like an oasis in the big city. Every time I enter, I feel like I can breathe fully, hear birds instead of cars, and see green trees instead of gray buildings.” (P15, Male, 34, Middle-income)*

#### Theme 2: Third places for social connection and community building (60%).

Parks emerged as crucial social infrastructure facilitating community formation—’third places’ [44] beyond home and work. Cross-class community formation was observed, with parks temporarily suspending income differences through shared activity groups.

*“Every morning, our exercise group meets at 6 am. We’re from different backgrounds—retired teachers, office workers, market vendors—but here we’re equal.” (P19, Male, 67, Low-income)*

#### Theme 3: Climate-specific accessibility challenges (73%).

Tropical climate conditions emerged as fundamental constraints, directly explaining the lowest-rated quantitative items. Climate barriers operated differentially by income: higher-income users arrived by air-conditioned private vehicle while lower-income users dependent on public transit faced extended heat exposure during travel.

*“During the hot season, I can only come very early in the morning or evening. The concrete gets so hot you can’t even sit on the benches.” (P03, Female, 29, High-income)*

#### Theme 4: Safety and maintenance concerns (67%).

Security issues and infrastructure deficiencies emerged as primary barriers with a pronounced gendered dimension. Of the 20 participants expressing safety concerns, 16 were women (80%). Inadequate evening lighting effectively constitutes a gendered exclusion from parks during cooler evening hours—the very hours when thermal barriers are reduced.

*“At night the lights aren’t bright enough, so I don’t dare come to exercise. Many women feel the same way.” (P25, Female, 27, Middle-income)*

#### Theme 5: Technology integration expectations (53%).

Participants expressed desires for digital infrastructure. Notably, of 16 participants expressing technology expectations, 13 (81%) were from households with incomes above 35,000 THB/month, suggesting a significant digital divide in technology expectations for parks. This finding cautions against prioritizing smart-park technology over basic quality improvements needed by all users.

#### Theme 6: Place attachment and ownership development (47%).

Regular users developed strong emotional bonds with specific parks, translating into stewardship behaviors consistent with Place Attachment Theory [33,36]. Place attachment was more commonly expressed by longer-term, higher-frequency users.

*“I feel like an owner here. When I see someone littering, I want to warn them because I think of this as my second home.” (P27, Male, 52, Middle-income)*

## Discussion

This study developed and validated a comprehensive integrated theoretical model for public park utilization and citizen satisfaction in tropical urban contexts—one that extends prior Bangkok-based research [49,50] through a broader multi-park sample and explicit equity analysis. The model’s explanatory power (R^2^ = 0.617–0.685) is high relative to typical R^2^ values in behavioral cross-sectional research [42]. This relatively high figure should be interpreted cautiously, however, as it partly reflects common-method variance from the use of self-reported predictors and outcomes and the conceptual proximity between some predictors and outcomes (see Limitations), rather than indicating inherent model superiority.

### Theoretical model validation

The Social Ecological Model’s contribution was most evident in the multi-level structure of findings. Although demographic control variables explained modest variance (ΔR^2^ = 0.022–0.027), park quality—an environmental-level determinant—explained the largest portion (β = 0.401–0.607), suggesting that individual behavior-change interventions, when they neglect environmental quality, may be insufficient on their own. Expectation-Disconfirmation Theory illuminated differential satisfaction across income groups: lower-income users’ systematically lower ratings likely reflect downwardly adjusted expectations shaped by historical exposure to lower-quality parks, a mechanism that can mask underlying inequity in aggregate statistics. Place Attachment Theory revealed that sustained usage and stewardship behaviors require parks to first meet basic quality thresholds, with implications for the continuity and consistency of park maintenance programs.

### Quality as the primary determinant

Quality’s emergence as the strongest predictor across all satisfaction outcomes represents one of the most policy-relevant findings. The data suggest that targeted quality improvements in existing parks are more strongly associated with satisfaction than expanding park coverage without attention to quality standards—a finding that directly complements Bangkok’s 15-minute park access initiative [50] by specifying what quality conditions parks must meet to realize health benefits. Priority quality dimensions include: physical infrastructure maintenance (equipment servicing, restroom cleanliness, path repairs); environmental quality enhancement (air quality, landscaping, noise management); and safety and security upgrades (lighting, security presence, emergency preparedness).

### Tropical climate adaptations as design necessities

Climate-specific features represent fundamental design requirements differing markedly from temperate guidelines. The two lowest-rated items—shaded walkways (M = 2.96) and nighttime lighting (M = 2.98)—both represent thermally and equity-driven design deficits. Drawing on established tropical urban design and microclimate literature [26], rather than on thresholds measured in the present study, the following parameters illustrate the magnitude of intervention these findings imply: heat protection systems (covered walkways providing on the order of 40% shade coverage; heat-resistant ground surfaces; cooling stations at roughly 150 m intervals); extended evening usage opportunities (pathway lighting of approximately 20 lux); and microclimate management (strategic canopy cover over an estimated 40–60% of active recreation zones; water features of around 500 m^2^, which the cited literature associates with ambient cooling of 1–3°C within 50 m [26]). These figures are offered as illustrative design targets to be calibrated locally, not as empirical results of this study.

The question of landscaping aesthetics is not trivial. Nassauer [58] identified a tension between ‘orderly’ designed landscapes and naturalistic ‘messy’ ones; this contrast is visible in Bangkok’s Benjakitti Park between its older grid section and newer forest park. Darnthamromgkul [53] found that Thai park users generally prefer visually tidy, maintained landscapes over naturalistic presentations, while still valuing natural canopy and biodiversity. Khew et al. [67] similarly found that preferences for aesthetic order and natural elements coexist in Singapore park users, reflecting broader Southeast Asian public sensibilities toward maintained yet biodiverse green spaces. Future park design should balance the ecological benefits of naturalistic planting with perceived cleanliness through thoughtful layout and edge management.

### Social health, equity, and policy implications

Parks’ function as ‘third places’ [44] supporting social capital formation has profound implications for mental health and community resilience. Social connections forged in parks are associated with reduced mortality risk [45] and improved immune function [46]. Suppakittpaisarn et al. [63,64] documented that both professionals and laypeople prefer denser, higher-quality green spaces. Jiang et al. [54] demonstrated a generalizable dose–response relationship between greenness exposure and mental health benefit. These findings collectively support treating parks as essential social and health infrastructure.

The significant socioeconomic gradient in satisfaction (η² = 0.039) demands equity-conscious policy responses. Bangkok’s 15-minute city park initiative represents an important access-equity commitment; our quality-first evidence complements this by specifying minimum quality standards that parks must meet to ‘count’ in coverage calculations. We recommend BMA integrate access and quality standards, with targeted investment in parks serving lower-income communities—currently disadvantaged by both longer travel distances and lower park quality.

Regarding environmental monitoring, the IQAir network of approximately 450 stations provides real-time PM2.5 data for Bangkok. Future park management should integrate this infrastructure by displaying real-time air quality information at park entrances and linking planting strategies to air quality data to prioritize particulate-filtering species in high-AQI zones, consistent with spatial-epidemiological approaches to targeting environmental health interventions [25].

### Limitations

This study has several limitations. The cross-sectional design limits causal inference; longitudinal studies are needed to establish temporal relationships. Sampling only current park users means non-user barriers are not captured, constraining conclusions about universal access. The potential for construct overlap between ‘Usage Patterns’ (predictor) and ‘Continued Usage Intention’ (outcome) may partly explain their high correlation (r = 0.644), though theoretical differentiation and temporal framing of items reduce this concern; a sensitivity analysis excluding Usage Patterns (S1 Table) confirmed that the principal conclusions are robust to this overlap. All measures are self-reported, introducing potential social desirability bias. Relatedly, the achieved sample was more educated and higher-income than the Bangkok population at large: 50.3% held at least a bachelor’s degree and roughly 33% reported household incomes above 35,000 THB/month, exceeding typical figures for the wider Bangkok Metropolitan Region and considerably exceeding national averages. Because participants were recruited on-site, this skew most likely reflects who currently uses the sampled parks—and who has the leisure time and transport access to do so—rather than sampling error alone. Two implications follow. First, the satisfaction levels reported here may be conservative with respect to the city’s most disadvantaged residents, who are under-represented among current users. Second, the socioeconomic gradients documented above—already significant within this comparatively advantaged sample—may understate the true magnitude of inequity in the broader population, which strengthens rather than weakens the study’s equity argument. Finally, findings from Bangkok may not generalize to all tropical megacities with different cultural, governance, or climatic contexts.

## Conclusions

This comprehensive mixed-methods investigation provides several contributions to environmental psychology, urban health research, and public policy. It delivers a validated integrated theoretical model combining the Social Ecological Model, Expectation-Disconfirmation Theory, and Place Attachment Theory for tropical urban park contexts, with explanatory power (R^2^ = 61.7–68.5%) that is high for cross-sectional behavioral research, though partly attributable to common-method effects (see Limitations). It identifies fundamental design requirements for tropical urban parks differing markedly from temperate guidelines; drawing on tropical urban design literature, it translates these findings into illustrative planning targets—covered walkways providing on the order of 40% shade coverage, canopy over roughly 40–60% of active zones, cooling stations at approximately 150 m intervals, pathway lighting of about 20 lux, and water features of around 500 m^2^ for microclimate cooling—offered as benchmarks to be calibrated locally rather than as thresholds measured here. It provides empirical evidence that targeted quality improvements are more strongly associated with satisfaction than quantity expansion—directly informing resource allocation in resource-constrained settings. It documents parks’ crucial function as ‘third places’ supporting social capital and community resilience. Critically, it indicates that park benefits are not equitably distributed, with lower-income residents facing compounded disadvantages in access, quality, and satisfaction. Addressing these inequities requires targeted investment in parks serving lower-income communities alongside the BMA’s citywide 15-minute access initiative.

## Supporting information

S1 File Complete questionnaire items (72-item instrument, English version). (PDF)

S2 File Factor analysis results (exploratory and confirmatory factor analysis). (PDF)

S3 File Demographic characteristics by urban zone and district. (PDF)

S4 File Complete seasonal variation analysis of park usage patterns. (PDF)

S5 File Qualitative interview guide. (PDF)

S6 File SPSS syntax for the statistical analyses. (PDF)

S7 File Anonymized interview transcript examples. (PDF)

S1 Table Sensitivity of the continued usage intention model to removal of usage patterns (standardized coefficients). (DOCX)
