# Supplementary material for: Public parks utilization and citizen satisfaction in Bangkok Metropolitan: An integrated theoretical model for tropical urban health
Source: PLoS One. 2026 Jul 27;21(7):e0354172. doi: 10.1371/journal.pone.0354172 (PMC13405312; doi:10.1371/journal.pone.0354172)
Supplement: S1 File — (PDF) [file pone.0354172.s001.pdf]

## S1 File. Complete Questionnaire Items (English Version)

*Public Park Utilization and Citizen Satisfaction in Bangkok Metropolitan. 5 sections, 72 items in total. This version reflects the instrument actually administered and matches the raw-data variable names (shown in brackets for traceability). It replaces the earlier S1, whose item counts (25 accessibility, 28 quality, etc.) did not match the dataset, the SPSS syntax, or the manuscript.*

### Section A: Demographics and Background (8 items)

**1.** Gender

**2.** Age (years)

**3.** Highest education level completed

**4.** Marital status

**5.** Occupation

**6.** Average monthly household income

**7.** Residential district

**8.** Distance from home to the park you use most often (km)

### Section B: Park Utilization (32 items)

*Items B1–B28 are rated on a 5-point Likert scale: 1 = Strongly disagree, 5 = Strongly agree.*

#### B.1 Accessibility (13 items)

Physical accessibility (5)

**1.** This park is located at a convenient distance from my home. *[acc_phys_distance]*

**2.** The routes to this park are safe to travel. *[acc_phys_safety]*

**3.** There are adequate shaded walkways on the way to the park. *[acc_phys_shade]*

**4.** This park is well connected to public transport. *[acc_phys_transit]*

**5.** Parking facilities at or near the park are sufficient. *[acc_phys_parking]*

Economic accessibility (4)

**6.** The travel cost to reach this park is affordable for me. *[acc_econ_cost]*

**7.** Any fees associated with using the park are reasonable. *[acc_econ_nofee]*

**8.** The time/opportunity cost of visiting this park is acceptable. *[acc_econ_time]*

**9.** I have adequate travel options for reaching this park. *[acc_econ_options]*

Social accessibility (4)

**10.** I feel safe and welcome using this park. *[acc_soc_safety]*

**11.** The park's opening hours suit my needs. *[acc_soc_hours]*

**12.** Information about the park (signage, rules, facilities) is easy to access. *[acc_soc_info]*

**13.** This park is suitable for people of all groups and ages. *[acc_soc_allages]*

### B.2 Quality (15 items)

Physical infrastructure (5)

**14.** Benches and seating are sufficient and in good condition. *[qual_phys_bench]*

**15.** Children's playground equipment is adequate and well maintained. *[qual_phys_playground]*

**16.** Exercise equipment is adequate and well maintained. *[qual_phys_exercise]*

**17.** Walking/cycling paths are in good condition. *[qual_phys_walkway]*

**18.** The landscape and grounds are attractive and well kept. *[qual_phys_landscape]*

Environmental quality (5)

**19.** Air quality in the park is good. *[qual_env_air]*

**20.** There is sufficient shaded area in the park. *[qual_env_shade]*

**21.** Ventilation/air flow in the park is good. *[qual_env_ventilation]*

**22.** The park supports diverse plants and wildlife (biodiversity). *[qual_env_biodiversity]*

**23.** Waste management and cleanliness are well handled. *[qual_env_waste]*

Safety and security (5)

**24.** Lighting in the park is adequate. *[qual_safe_lighting]*

**25.** Access control to the park is appropriate. *[qual_safe_access]*

**26.** Visibility/sight lines throughout the park are good. *[qual_safe_visibility]*

**27.** Security guards/personnel are adequate. *[qual_safe_guard]*

**28.** Park staff provide good care and service. *[qual_safe_staff]*

### B.3 Usage Patterns (4 items)

**1.** How often have you visited this park in the past 4 weeks? (visits per week) *[Usage1 / ความถี่]*

**2.** On average, how long do you stay per visit? (hours) *[Usage2 / ระยะเวลา]*

**3.** How many different types of activities do you usually do per visit? (select all that apply) *[Usage3 / จำนวนกิจกรรม]*

**4.** How regular/consistent is your park use? *[Usage4 / ความสม่ำเสมอ]*

## Section C: Satisfaction (27 items)

*All items rated 1 = Strongly disagree/Very dissatisfied to 5 = Strongly agree/Very satisfied.*

### C.1 Overall Satisfaction (3 items)

**1.** Overall, I am satisfied with this park. *[sat_ov_general]*

**2.** My overall experience using this park is positive. *[sat_ov_experience]*

**3.** I enjoy spending time in this park. *[sat_ov_enjoyment]*

### C.2 Domain-Specific Satisfaction (15 items)

Facilities (3)

**4.** I am satisfied with the restroom facilities. *[sat_fac_restroom]*

**5.** I am satisfied with drinking-water facilities. *[sat_fac_water]*

**6.** I am satisfied with parking facilities. *[sat_fac_parking]*

Safety (3)

**7.** I am satisfied with protection against crime. *[sat_safe_crime]*

**8.** I am satisfied with accident prevention/safety. *[sat_safe_accident]*

**9.** I am satisfied with safety for children. *[sat_safe_children]*

Cleanliness and maintenance (3)

**10.** I am satisfied with overall cleanliness. *[sat_clean_general]*

**11.** I am satisfied with the upkeep of gardens/greenery. *[sat_clean_garden]*

**12.** I am satisfied with repair and maintenance of facilities. *[sat_clean_repair]*

Atmosphere and environment (3)

**13.** I am satisfied with the relaxing atmosphere. *[sat_atm_relax]*

**14.** I am satisfied with the landscape/scenery. *[sat_atm_landscape]*

**15.** I am satisfied with the air/freshness. *[sat_atm_air]*

Access and travel convenience (3)

**16.** I am satisfied with the convenience of travelling to the park. *[sat_conv_travel]*

**17.** I am satisfied with public-transport access. *[sat_conv_transit]*

**18.** I am satisfied with signage/wayfinding. *[sat_conv_signage]*

### C.3 Continued Usage Intention (9 items)

Repeat use (3)

**19.** I intend to return to this park. *[int_repeat_return]*

**20.** This park is among my first choices for recreation. *[int_repeat_first]*

**21.** I intend to continue using this park regularly. *[int_repeat_continue]*

Recommendation (3)

**22.** I would recommend this park to friends. *[int_rec_friends]*

**23.** I would speak positively about this park to others. *[int_rec_positive]*

**24.** I would share information about this park with others. *[int_rec_share]*

Loyalty / attachment (3)

**25.** I feel attached to this park. *[int_loy_attach]*

**26.** This park is part of my way of life. *[int_loy_partoflife]*

**27.** I would help protect and care for this park. *[int_loy_protect]*

## Section D: Additional Context (4 items)

**1.** The park you use most often

**2.** Time of day you usually visit

**3.** Main mode of transport to the park

**4.** Who you usually come with

## Section E: Suggestions (1 item, open-ended)

**1.** Suggestions for improving the park (open-ended).

*Summary of item counts: Demographics 8 + Park Utilization 32 (Accessibility 13, Quality 15, Usage 4) + Satisfaction 27 (Overall 3, Domain 15, Continued Usage Intention 9) + Additional 4 + Suggestions 1 = 72 items. These counts match the SPSS composites (S6) and the construct definitions in the manuscript and Table 1/2.*
