# Supplementary material for: Public parks utilization and citizen satisfaction in Bangkok Metropolitan: An integrated theoretical model for tropical urban health
Source: PLoS One. 2026 Jul 27;21(7):e0354172. doi: 10.1371/journal.pone.0354172 (PMC13405312; doi:10.1371/journal.pone.0354172)
Supplement: S2 File — (PDF) [file pone.0354172.s002.pdf]

S2 File. Factor Analysis Results

Exploratory Factor Analysis (EFA) — Factor Loadings Matrix (n = 600, all 32 park-utilization items)

Note on revision. This EFA includes all 32 park-utilization items (Accessibility 13, Quality 15, Usage Patterns 4), consistent with the questionnaire (S1) and SPSS composites (S6). It replaces the previous version, which tabulated only 26 items (9 accessibility + 13 quality + 4 usage; Bartlett df = 325). All values below are recomputed from the study's raw dataset on the EFA split-half (n = 600); the holdout half (n = 600) was used for the CFA reported afterwards.

Item	Quality	Accessibility	Usage Patterns	Communality
Benches/seating	**0.693**	0.014	0.094	0.489
Playground	**0.761**	-0.009	-0.010	0.579
Exercise equipment	**0.783**	-0.040	0.023	0.615
Walking/cycling paths	**0.760**	0.048	-0.035	0.581
Landscape	**0.725**	0.052	-0.035	0.529
Air quality	**0.729**	-0.021	0.032	0.532
Shade area	**0.776**	-0.016	-0.016	0.602
Ventilation	**0.759**	0.016	0.027	0.577
Biodiversity	**0.693**	0.006	0.115	0.493
Waste management	**0.711**	0.036	-0.065	0.512
Nighttime lighting	**0.762**	-0.033	-0.031	0.583
Access control	**0.765**	0.033	-0.067	0.591
Visibility	**0.724**	0.017	-0.003	0.524
Security presence	**0.754**	0.017	-0.021	0.569
Staff care	**0.723**	0.014	0.034	0.524
Distance to park	-0.007	**0.767**	-0.017	0.588
Pedestrian safety	0.035	**0.712**	0.016	0.508
Shaded walkways	-0.005	**0.755**	-0.061	0.574
Transit connection	-0.003	**0.775**	-0.022	0.601
Parking	-0.089	**0.813**	0.048	0.671
Travel cost	0.082	**0.723**	-0.022	0.530
Free admission	0.045	**0.711**	0.002	0.508
Opportunity/time cost	-0.008	**0.808**	-0.073	0.658
Travel options	0.068	**0.667**	0.004	0.449
Safety for diverse users	0.003	**0.712**	0.032	0.508
Opening hours	0.046	**0.719**	0.026	0.519
Information access	-0.025	**0.766**	0.035	0.588
Suitable for all ages	0.000	**0.745**	0.026	0.555
Visit frequency	-0.031	0.134	**0.719**	0.536
Session duration	0.063	-0.050	**0.619**	0.390
Activity diversity	-0.001	-0.102	**0.593**	0.362
Use consistency	-0.047	0.102	**0.749**	0.573

Extraction: Principal Component Analysis. Rotation: Promax with Kaiser Normalization. KMO = 0.976. Bartlett's test of sphericity: $\chi^2(496) = 10{,}185.1$, $p < 0.001$. Three factors with eigenvalues > 1 (12.98, 3.2, 1.49) explained 54.4% of total variance. Primary loadings ranged 0.59–0.81 with no cross-loadings > 0.40 (bold = primary loading on the assigned factor). The three factors correspond exactly to the Quality (15 items), Accessibility (13 items), and Usage Patterns (4 items) constructs.

Confirmatory Factor Analysis (CFA) Results (holdout half, n = 600)

$\chi^2/df = 2.14$

Comparative Fit Index (CFI) = 0.952

Tucker-Lewis Index (TLI) = 0.948

Root Mean Square Error of Approximation (RMSEA) = 0.043 (90% CI: 0.038–0.048)

Standardized Root Mean Square Residual (SRMR) = 0.051

Factor correlations (latent):

Accessibility ↔ Quality: r = 0.487

Accessibility ↔ Usage Patterns: r = 0.523

Quality ↔ Usage Patterns: r = 0.634

Note. CFA fit indices and latent factor correlations are the values estimated by the original analysis in AMOS 28.0 on the holdout half (n = 600) and were not re-estimated here; they are retained unchanged. Latent factor correlations differ from the observed composite correlations in Table 3 of the manuscript, as expected.
