# Supplementary material for: Public parks utilization and citizen satisfaction in Bangkok Metropolitan: An integrated theoretical model for tropical urban health
Source: PLoS One. 2026 Jul 27;21(7):e0354172. doi: 10.1371/journal.pone.0354172 (PMC13405312; doi:10.1371/journal.pone.0354172)
Supplement: S3 File — (PDF) [file pone.0354172.s003.pdf]

## S3 File. Demographic Characteristics by Urban Zone and District

*Distribution across the three-zone stratified random sample (N = 1,200)*

**Sampling design.** Bangkok's 50 districts were stratified into three urban zones by population density and urban development (central, intermediate, suburban). Five districts were randomly selected from each zone (15 districts total); two parks were randomly selected per district (30 parks total); and 40 respondents were systematically sampled per park, yielding a balanced design of 400 respondents per zone and 80 per district. All figures below are computed directly from the study's raw dataset.

| Zone / District | n | % | Mean age (SD) | Female % | Bachelor's + % | High income % (>50,000) |
|---|---|---|---|---|---|---|
| **Central zone** | **400** | **33.3** | **35.4 (12.7)** | **59.8** | **52.7** | **11.2** |
| Phra Nakhon | 80 | 6.7 | 34.6 (11.6) | 58.8 | 53.2 | 11.2 |
| Bang Rak | 80 | 6.7 | 35.4 (12.5) | 58.8 | 55.7 | 15.0 |
| Pathum Wan | 80 | 6.7 | 34.8 (13.8) | 56.2 | 50.6 | 5.0 |
| Watthana | 80 | 6.7 | 36.0 (12.6) | 58.8 | 50.0 | 13.8 |
| Sathon | 80 | 6.7 | 36.3 (13.2) | 66.2 | 53.8 | 11.2 |
| **Intermediate zone** | **400** | **33.3** | **36.5 (13.1)** | **55.5** | **45.6** | **11.2** |
| Yan Nawa | 80 | 6.7 | 38.9 (12.9) | 53.8 | 43.8 | 10.0 |
| Khlong Toei | 80 | 6.7 | 35.0 (13.2) | 51.2 | 44.9 | 12.5 |
| Bang Kapi | 80 | 6.7 | 36.3 (13.2) | 58.8 | 43.0 | 16.2 |
| Chatuchak | 80 | 6.7 | 36.0 (13.4) | 56.2 | 45.6 | 8.8 |
| Huai Khwang | 80 | 6.7 | 36.4 (12.7) | 57.5 | 50.6 | 8.8 |
| **Suburban zone** | **400** | **33.3** | **35.6 (12.0)** | **59.8** | **52.8** | **14.0** |
| Prawet | 80 | 6.7 | 35.6 (13.1) | 52.5 | 57.5 | 8.8 |
| Thung Khru | 80 | 6.7 | 36.1 (11.0) | 60.0 | 57.1 | 12.5 |
| Bang Sue | 80 | 6.7 | 37.9 (12.2) | 57.5 | 50.0 | 11.2 |
| Lat Phrao | 80 | 6.7 | 34.0 (10.4) | 62.5 | 45.6 | 17.5 |
| Bang Khae | 80 | 6.7 | 34.5 (12.7) | 66.2 | 53.8 | 20.0 |
| **Total** | **1200** | **100.0** | **35.9 (12.6)** | **58.3** | **50.3** | **12.2** |

**Note.** Bachelor's+ % is computed over valid education responses (n = 1,182; 18 missing). High income = reported monthly household income above 50,000 THB. Mean age 35.9 (SD 12.6), 58.3% female, 50.3% bachelor's degree or above, and the income distribution all match the values reported in the main manuscript. Age groups (from continuous age): 18–30 years n = 466, 31–50 years n = 554, 51+ years n = 180.

**Correction note.** This table replaces the previously supplied S3, which listed 16 districts with an unbalanced 600/400/200 allocation and labelled the strata as "socioeconomic (high/medium/low SES)." That version did not correspond to the study's raw data or to the sampling design (15 districts, balanced 400 per zone). The strata are urban-density zones, not SES strata: the suburban zone in fact has the highest proportion of high-income respondents (14.0%), so a high/medium/low SES labelling is not supported by the

data. The manuscript Methods wording has accordingly been revised from “three SES strata (high, medium, low)” to “three urban-density zones (central, intermediate, suburban)”; socioeconomic comparisons are handled separately via the income-group analysis (Table 5).
