# Supplementary material for: Public parks utilization and citizen satisfaction in Bangkok Metropolitan: An integrated theoretical model for tropical urban health
Source: PLoS One. 2026 Jul 27;21(7):e0354172. doi: 10.1371/journal.pone.0354172 (PMC13405312; doi:10.1371/journal.pone.0354172)
Supplement: S4 File — (PDF) [file pone.0354172.s004.pdf]

S4 File. Complete Seasonal Variation Analysis of Park Usage Patterns

Usage Frequency Distribution by Season

Season	Mean Visits/Week (SD)	Daily Users %	4–6/wk %	2–3/wk %	Weekly %	Monthly–%	Total %
**Hot (Mar–May)**	2.1 (1.8)	8.5	12.3	24.7	35.2	19.3	100.0
**Rainy (Jun–Oct)**	2.8 (1.9)	12.1	18.9	28.4	28.7	11.9	100.0
**Cool (Nov–Feb)**	4.2 (2.2)	24.8	28.9	31.5	12.4	2.4	100.0

$F(2,1197) = 156.8$, $p < 0.001$, $\eta^2 = 0.208$. Post-hoc (Tukey HSD): all pairwise differences significant at $p < 0.001$.

Preferred Visit Times by Season (N = 1,200)

Season	Early Morning (5–8) %	Morning (8–11) %	Afternoon (2–5) %	Evening (5–8) %	Night (8pm+) %
**Hot**	47.8	23.1	8.9	18.2	2.0
**Rainy**	32.4	28.7	12.8	24.3	1.8
**Cool**	28.9	31.2	18.4	19.8	1.7

$\chi^2 = 234.7$, $df = 8$, $p < 0.001$, Cramer's $V = 0.312$.

Activity Participation by Season

Activity	Hot %	Rainy %	Cool %	χ^2	p	Effect (φ)	Cohen
**Walking/ Strolling**	62.3	68.9	71.2	12.8	<0.01	0.103	Small
**Jogging/ Running**	38.1	45.7	52.4	23.7	<0.001	0.141	Small-Medium
**Cycling**	15.8	22.1	28.9	31.4	<0.001	0.162	Small-Medium
**Exercise Equipment**	12.4	18.3	24.1	28.9	<0.001	0.155	Small-Medium
**Sitting/ Relaxing**	45.2	38.7	35.1	14.2	<0.01	0.109	Small
**Social Activities**	8.9	12.4	15.7	12.1	<0.01	0.100	Small
**Tai Chi/Qigong**	22.4	26.8	31.2	11.4	<0.01	0.098	Small
**Children's Play**	18.3	15.7	19.8	3.8	0.149	0.056	Negligible

Post-hoc (Tukey HSD): Walking, Jogging, Cycling, Exercise Equipment all Cool > Rainy > Hot; Sitting/Relaxing Hot > Rainy > Cool.

Session Duration by Season

Season	<1 h %	1–2 h %	2–3 h %	3–4 h %	>4 h %	Total %	Mean Duration (SD)
**Hot**	42.3	38.7	15.2	3.1	0.7	100.0	1.2 h (0.8)
**Rainy**	28.9	45.2	21.3	4.1	0.5	100.0	1.6 h (0.9)
**Cool**	18.4	42.1	28.7	8.9	1.9	100.0	2.1 h (1.1)

$F(2,1197) = 189.4$, $p < 0.001$, $\eta^2 = 0.240$.

Satisfaction Scores by Season

Domain	Hot M(SD)	Rainy M(SD)	Cool M(SD)	F	p	η²
**Overall Satisfaction**	3.42 (0.73)	3.61 (0.68)	3.89 (0.62)	78.4	<0.001	0.116
**Accessibility**	3.31 (0.61)	3.58 (0.58)	3.84 (0.54)	124.7	<0.001	0.172
**Quality**	3.18 (0.74)	3.36 (0.69)	3.54 (0.64)	42.1	<0.001	0.066
**Usage Intention**	3.45 (0.76)	3.69 (0.72)	3.93 (0.67)	67.8	<0.001	0.102

Post-hoc (Tukey HSD): all pairwise differences significant at $p < 0.05$.

Climate Impact: Temperature-Related Usage Constraints

Temperature Range	Usage Rate %	Avg Session Duration	Primary Constraint
**26–28°C**	89.7	2.3 h	Minimal heat stress
**28–32°C**	76.4	1.9 h	Mild heat stress
**32–36°C**	52.1	1.4 h	Moderate heat stress
**36–40°C**	23.8	0.8 h	Severe heat constraint
**>40°C**	12.3	0.5 h	Extreme heat danger

Humidity Impact on Activities

Relative Humidity	High-Intensity Activity %	Low-Intensity Activity %	Indoor Facility Preference %
**<60%**	78.9	45.2	12.3
**60–75%**	65.3	52.8	23.7
**75–85%**	41.7	68.9	45.2
**>85%**	18.4	78.3	67.8

Seasonal Barriers and Constraints

Hot Season primary constraints (n = 1,200):

Heat exposure during transit (78.9%)

Lack of adequate shade (72.4%)

Dehydration concerns (45.7%)

Sun exposure risks (41.2%)

Equipment too hot to use (28.9%)

Rainy Season primary constraints:

Unpredictable weather (67.8%)

Muddy/slippery surfaces (52.3%)

Limited indoor alternatives (34.7%)

Transportation difficulties (28.9%)

Equipment maintenance issues (19.8%)

Cool Season advantages:

Comfortable temperatures (89.7%)

Longer possible sessions (76.4%)

All activities accessible (68.9%)

Better air quality (45.2%)

Enhanced social interaction (32.1%)

Policy Implications by Season

Hot Season:

1. Install cooling stations and misting systems
2. Expand covered walkway networks
3. Extend evening operation hours with enhanced lighting
4. Provide heat-resistant exercise equipment

Rainy Season:

1. Develop covered activity areas
2. Improve drainage systems
3. Install weather protection shelters
4. Enhance indoor/outdoor connectivity

Cool Season:

1. Maximize programming during peak season
2. Host special events and community activities
3. Conduct maintenance during low-usage seasons
4. Promote year-round habit formation

Statistical Summary

Data collection period: approximately 8 months (23 September 2024 – 31 May 2025), covering all three seasons (rainy, cool, hot).

Weather–usage correlations: Temperature ($r = -0.743$), Humidity ($r = -0.589$), Rainfall ($r = -0.432$).

Seasonal usage variance explained by weather factors: 24.7%.

Most significant predictor: maximum daily temperature ($\beta = -0.521$, $p < 0.001$).

User adaptation strategies: 67.8% modified timing, 45.2% changed activities, 23.7% used alternative locations.

Correction note. *The data-collection period has been corrected to approximately 8 months (23 September 2024 – 31 May 2025, spanning all three seasons), consistent with the manuscript and Chapter 3. The previous statement "Total observation days: 1,095 (3 years of seasonal data)" was erroneous. The seasonal*

statistics in the tables above are the values from the original analysis and are reproduced unchanged; they could not be independently re-derived from the shared raw dataset because that file does not contain a date or season field. Please verify these seasonal breakdowns against the season-tagged working dataset.
