# Supplementary material for: Public parks utilization and citizen satisfaction in Bangkok Metropolitan: An integrated theoretical model for tropical urban health
Source: PLoS One. 2026 Jul 27;21(7):e0354172. doi: 10.1371/journal.pone.0354172 (PMC13405312; doi:10.1371/journal.pone.0354172)
Supplement: S5 File — (PDF) [file pone.0354172.s005.pdf]

S5 File. Qualitative Interview Guide

English Version

Introduction: This interview will take approximately 45–60 minutes. We want to understand your experiences, feelings, and opinions about using public parks. The interview will be audio-recorded, and all information will be kept confidential.

Section 1: Park Experience

1. Can you tell me about your relationship with this park?

2. How did you first discover this park?

3. Describe a typical visit to this park for me.

4. What do you enjoy most about coming here?

5. What challenges or difficulties do you experience when using this park?

6. How does your park usage change across different seasons?

Section 2: Quality Assessment

7. How would you describe the overall quality of this park?

8. What aspects work well for you? What doesn’t work well?

9. How does this park compare to other parks you’ve used?

10. Have you noticed any changes in this park over time?

Section 3: Place Attachment

11. What feelings does this park evoke for you?

12. Do you feel any sense of connection or ownership toward this park?

13. What memories do you associate with this place?

14. Does this park have any special meaning for you?

Section 4: Improvement Recommendations

15. If you could make three changes to improve this park, what would they be?

16. How should limited improvement budgets be prioritized?

17. What would make this park more accessible to people like you?

18. Do you have any other suggestions or comments?

Note (correction). *The interview duration is stated as 45–60 minutes, consistent with the methodology (Chapter 3, §3.3.3) and the manuscript. An earlier version of this guide stated “60–75 minutes,” which was an error.*
