# Supplementary material for: Public parks utilization and citizen satisfaction in Bangkok Metropolitan: An integrated theoretical model for tropical urban health
Source: PLoS One. 2026 Jul 27;21(7):e0354172. doi: 10.1371/journal.pone.0354172 (PMC13405312; doi:10.1371/journal.pone.0354172)
Supplement: S6 File — (PDF) [file pone.0354172.s006.pdf]

```
* SPSS Syntax for Public Park Utilization Analysis.
* Author: Wichian Chanthanet.
* Date: June 2025 (corrected).
* ----------------------------------------------------------------------------.
* CORRECTION NOTE:
*   Two composite definitions were corrected to match the questionnaire
*   structure and the manuscript (verified against the raw data, where the
*   reproduced Cronbach's alpha values match Table 1/2 exactly):
*     - Overall_Satisfaction now uses OverSat1 to OverSat3   (3 items, was 5).
*     - Usage_Intention      now uses Intention1 to Intention9 (9 items, was 3).
*   Item counts: Accessibility 13, Quality 15, Usage 4,
*                Overall Satisfaction 3, Domain Satisfaction 15,
*                Continued Usage Intention 9  (questionnaire total = 72 items).
* ----------------------------------------------------------------------------.

* Set working directory and import data.
FILE HANDLE project /NAME='C:\ParkStudy\Data\'.
GET DATA
 /TYPE=XLSX
 /FILE='project\ParkData_Final.xlsx'
 /SHEET=name 'Survey_Data'
 /CELLRANGE=full
 /READNAMES=on.

* Data Preparation.
COMPUTE Age_Group = 1.
IF (Age >= 31 AND Age <= 50) Age_Group = 2.
IF (Age >= 51) Age_Group = 3.
VALUE LABELS Age_Group 1 '18-30 years' 2 '31-50 years' 3 '51+ years'.

COMPUTE Income_Group = 1.
IF (Income >= 25001 AND Income <= 50000) Income_Group = 2.
IF (Income > 50000) Income_Group = 3.
VALUE LABELS Income_Group 1 'Low' 2 'Medium' 3 'High'.

* Compute composite scores.
COMPUTE Accessibility       = MEAN(Access1 to Access13).
COMPUTE Quality             = MEAN(Quality1 to Quality15).
COMPUTE Usage_Patterns      = MEAN(Usage1 to Usage4).
COMPUTE Overall_Satisfaction = MEAN(OverSat1 to OverSat3).
COMPUTE Domain_Satisfaction  = MEAN(DomSat1 to DomSat15).
COMPUTE Usage_Intention      = MEAN(Intention1 to Intention9).

* Reliability Analysis.
RELIABILITY
 /VARIABLES=Access1 to Access13
 /SCALE('Accessibility') ALL
 /MODEL=ALPHA
 /STATISTICS=DESCRIPTIVE SCALE CORR COV
 /SUMMARY=MEANS VARIANCE COV CORR.
RELIABILITY
 /VARIABLES=Quality1 to Quality15
 /SCALE('Quality') ALL
 /MODEL=ALPHA
 /STATISTICS=DESCRIPTIVE SCALE CORR COV
 /SUMMARY=MEANS VARIANCE COV CORR.
RELIABILITY
 /VARIABLES=Usage1 to Usage4
 /SCALE('Usage Patterns') ALL
 /MODEL=ALPHA
 /STATISTICS=DESCRIPTIVE SCALE CORR COV
 /SUMMARY=MEANS VARIANCE COV CORR.
RELIABILITY
 /VARIABLES=OverSat1 to OverSat3
 /SCALE('Overall Satisfaction') ALL
 /MODEL=ALPHA.
RELIABILITY
 /VARIABLES=DomSat1 to DomSat15
 /SCALE('Domain Satisfaction') ALL
 /MODEL=ALPHA.
RELIABILITY
 /VARIABLES=Intention1 to Intention9
 /SCALE('Continued Usage Intention') ALL
 /MODEL=ALPHA.

* Descriptive Statistics.
DESCRIPTIVES VARIABLES=Age Accessibility Quality Usage_Patterns
 Overall_Satisfaction Domain_Satisfaction Usage_Intention
```

```
 /STATISTICS=MEAN STDDEV MIN MAX.

FREQUENCIES VARIABLES=Gender Education Income_Group Age_Group
 /STATISTICS=MODE
 /ORDER=ANALYSIS.

* Correlation Analysis.
CORRELATIONS
 /VARIABLES=Accessibility Quality Usage_Patterns Overall_Satisfaction
 Domain_Satisfaction Usage_Intention
 /PRINT=TWOTAIL NOSIG
 /STATISTICS DESCRIPTIVES.

* Factor Analysis - EFA.
* NOTE: EFA was conducted on ALL 32 park-utilization items
*       (Accessibility 13 + Quality 15 + Usage 4) on the EFA split-half
*       (n = 600). The solution is a clean three-factor structure
*       (KMO = 0.976; Bartlett df = 496; 54.4% total variance;
*       primary loadings 0.59-0.81; no cross-loadings > 0.40),
*       matching S2 File and the manuscript.
FACTOR
 /VARIABLES Access1 to Access13 Quality1 to Quality15 Usage1 to Usage4
 /MISSING LISTWISE
 /ANALYSIS Access1 to Access13 Quality1 to Quality15 Usage1 to Usage4
 /PRINT INITIAL CORRELATION SIG KMO AIC EXTRACTION ROTATION
 /FORMAT SORT BLANK(.40)
 /PLOT EIGEN ROTATION
 /CRITERIA MINEIGEN(1) ITERATE(25)
 /EXTRACTION PC
 /CRITERIA ITERATE(25) DELTA(0)
 /ROTATION PROMAX(4).

* Hierarchical Multiple Regression - Overall Satisfaction.
REGRESSION
 /MISSING LISTWISE
 /STATISTICS COEFF OUTS CI(95) R ANOVA CHANGE ZPP
 /CRITERIA=PIN(.05) POUT(.10)
 /NOORIGIN
 /DEPENDENT Overall_Satisfaction
 /METHOD=ENTER Gender Age Education Income
 /METHOD=ENTER Accessibility Quality Usage_Patterns.

* Hierarchical Multiple Regression - Domain Satisfaction.
REGRESSION
 /MISSING LISTWISE
 /STATISTICS COEFF OUTS CI(95) R ANOVA CHANGE ZPP
 /CRITERIA=PIN(.05) POUT(.10)
 /NOORIGIN
 /DEPENDENT Domain_Satisfaction
 /METHOD=ENTER Gender Age Education Income
 /METHOD=ENTER Accessibility Quality Usage_Patterns.

* Hierarchical Multiple Regression - Usage Intention.
REGRESSION
 /MISSING LISTWISE
 /STATISTICS COEFF OUTS CI(95) R ANOVA CHANGE ZPP
 /CRITERIA=PIN(.05) POUT(.10)
 /NOORIGIN
 /DEPENDENT Usage_Intention
 /METHOD=ENTER Gender Age Education Income
 /METHOD=ENTER Accessibility Quality Usage_Patterns.

* ANOVA for demographic differences.
ONEWAY Overall_Satisfaction BY Age_Group
 /STATISTICS DESCRIPTIVES HOMOGENEITY
 /PLOT MEANS
 /MISSING ANALYSIS
 /POSTHOC=TUKEY ALPHA(0.05).
ONEWAY Overall_Satisfaction BY Income_Group
 /STATISTICS DESCRIPTIVES HOMOGENEITY
 /PLOT MEANS
 /MISSING ANALYSIS
 /POSTHOC=TUKEY ALPHA(0.05).

T-TEST GROUPS=Gender(0 1)
 /MISSING=ANALYSIS
 /VARIABLES=Overall_Satisfaction Domain_Satisfaction Usage_Intention
 /CRITERIA=CI(.95).
```

```
* Common Method Bias Testing - Harman's Single Factor Test.
FACTOR
 /VARIABLES Access1 to Access13 Quality1 to Quality15 Usage1 to Usage4
 OverSat1 to OverSat3 DomSat1 to DomSat15 Intention1 to Intention9
 /MISSING LISTWISE
 /ANALYSIS Access1 to Access13 Quality1 to Quality15 Usage1 to Usage4
 OverSat1 to OverSat3 DomSat1 to DomSat15 Intention1 to Intention9
 /PRINT INITIAL EXTRACTION
 /CRITERIA FACTORS(1) ITERATE(25)
 /EXTRACTION PC.
```
