# Supplementary material for: Public parks utilization and citizen satisfaction in Bangkok Metropolitan: An integrated theoretical model for tropical urban health
Source: PLoS One. 2026 Jul 27;21(7):e0354172. doi: 10.1371/journal.pone.0354172 (PMC13405312; doi:10.1371/journal.pone.0354172)
Supplement: S7 File — (PDF) [file pone.0354172.s007.pdf]

S7 File: Anonymized Interview Transcript Examples

Overview

This supplementary file provides representative excerpts from the 30 in-depth interviews conducted as part of the qualitative phase. All transcripts have been anonymized following ethical protocols, with identifying information removed and participant codes assigned. Complete transcripts are available upon request following the data-sharing protocol outlined in the main manuscript.

Interview Methodology Reminder

Total interviews: 30 participants

Duration: 45–60 minutes per interview

Language: Thai (translated to English for this document)

Setting: Quiet locations near parks or participants' homes

Recording: Audio-recorded with permission

Transcription: Verbatim transcription with cultural context notes

Theme 1: Parks as Urban Oases and Psychological Restoration Spaces

Interview Excerpt 1.1 (P15, Male, 34, Middle-income, 4 visits/week)

Interviewer: Can you tell me about your relationship with this park?

P15: [Long pause] The park is like... how can I say... It’s like an oasis in this big city. Every time I enter here, I feel like I can breathe fully again. In Bangkok we live surrounded by concrete buildings, cars everywhere, noise all the time. But when I come here, I hear birds singing instead of car horns. I see green trees instead of gray buildings. It’s like entering a different world completely.

Interviewer: What does that mean for you personally?

P15: For my mental health, it’s very important. When I’m stressed from work or family problems, this is where I come to find peace. The trees, the open space, the fresh air — it’s like therapy, but it’s free.

Interview Excerpt 1.2 (P08, Female, 41, Low-income, 2 visits/week)

P08: Every day I work in the factory, standing for 10 hours. When I come here after work, even for just 30 minutes, it’s like washing my soul clean. This park is like medicine for people like us who can’t afford real therapy or a vacation. Here, rich and poor people can both find peace under the same sky.

Theme 2: Third Places for Social Connection and Community Building

Interview Excerpt 2.1 (P22, Female, 52, Middle-income, 5 visits/week)

P22: I met many new friends here. We became like a family, caring for each other, sharing life stories. In Bangkok it's hard to know your neighbors, but here we have a real community. When my husband was in the hospital last year, these friends visited, brought food, and took care of me.

Interview Excerpt 2.2 (P19, Male, 67, Low-income, Daily visits)

P19: Every morning, 6 AM sharp, we meet at the pavilion. We're from different backgrounds — retired teachers, office workers, market vendors, security guards — but here we're equal. This park created bonds that wouldn't exist otherwise.

Theme 3: Climate-Specific Accessibility Challenges

Interview Excerpt 3.1 (P12, Male, 41, Low-income, 2 visits/week)

P12: The main problem is walking here in the hot sun. My apartment is only 800 meters away, but by the time I arrive I'm already exhausted and sweating. During the hot season I can only come very early morning or evening. The concrete gets so hot you can't sit on the benches.

Interview Excerpt 3.2 (P03, Female, 29, High-income, 3 visits/week)

P03: I have to plan everything around the heat. If it's over 35 degrees, I only come for the evening yoga class. More covered walkways and some indoor spaces with fans for extremely hot days would help.

Theme 4: Safety and Maintenance Concerns

Interview Excerpt 4.1 (P25, Female, 27, Middle-income, 3 visits/week)

P25: During the daytime I feel quite safe. But at night the lights aren't bright enough. I don't dare come alone to exercise after sunset, even though that would be cooler. Many women feel the same way.

Interview Excerpt 4.2 (P14, Male, 55, Medium-income, 4 visits/week)

P14: The exercise equipment breaks often and takes weeks to repair. When facilities are broken, we feel like the government doesn't care about public health. Good maintenance shows respect for citizens.

Theme 5: Technology Integration Expectations

Interview Excerpt 5.1 (P06, Female, 23, High-income, 3 visits/week)

P06: Free WiFi would be great. Maybe apps to track our running routes and QR codes with information about plants and birds. Digital signs showing air quality, temperature, and UV index would help us plan activities safely.

Interview Excerpt 5.2 (P11, Male, 35, High-income, 4 visits/week)

P11: Online booking for sports courts and digital maps showing crowded versus quiet spots would help — but it should supplement nature, not replace it. The point is still fresh air, green space, and human connection.

Theme 6: Place Attachment and Ownership Development

Interview Excerpt 6.1 (P27, Male, 52, Middle-income, Daily visits)

P27: I feel like a part-owner here. When I see someone littering, I want to warn them because I think of this as my second home. I've watched the trees grow from small saplings to big shade providers.

Interview Excerpt 6.2 (P21, Female, 58, Medium-income, 4 visits/week)

P21: So many precious memories — first bringing my children here, later bringing my grandchildren to the same playground. This park holds three generations of our family story.

Methodological Notes

Anonymization Protocol

- All proper names (people, specific locations) removed
- Specific occupational details generalized
- Exact ages rounded to protect identity
- Direct quotes modified minimally to prevent identification

Interview Schedule Summary

Total interviews: 30. Duration: 45–60 minutes each (approximately 22.5–30 hours in total). Language distribution: 28 Thai, 2 English. Gender distribution: 12 female, 18 male. Age range: 19–67 years (mean 41.2 years). Income distribution: 9 low, 13 middle, 8 high income. Geographic coverage: 18 of the 30 study parks represented.

Note (correction). *Interview duration is reported as 45–60 minutes per interview, consistent with the methodology (Chapter 3, §3.3.3), the interview guide (S5), and the manuscript. Earlier figures in this file ("45–90 minutes, mean 67 minutes" and "total 33.5 hours") were erroneous: 30 interviews of 45–60 minutes total approximately 22.5–30 hours, not 33.5 hours.*
