# Supplementary material for: Public parks utilization and citizen satisfaction in Bangkok Metropolitan: An integrated theoretical model for tropical urban health
Source: PLoS One. 2026 Jul 27;21(7):e0354172. doi: 10.1371/journal.pone.0354172 (PMC13405312; doi:10.1371/journal.pone.0354172)
Supplement: S1 Table — (DOCX) [file pone.0354172.s008.docx]

**S1 Table. Sensitivity of the Continued Usage Intention model to removal of Usage Patterns (standardized coefficients).**

| **Predictor / statistic** | **Full model** | **Reduced model (Usage Patterns removed)** |
| --- | --- | --- |
| Accessibility | 0.18 | 0.31 |
| Quality | 0.40 | 0.49 |
| Usage Patterns | 0.35 | — (removed) |
| R² | 0.61 | 0.53 |

*Note. Full-model coefficients are standardized estimates from the three-predictor model (accessibility, quality, usage patterns) without demographic controls; reduced-model coefficients omit usage patterns. Removing usage patterns reduces R² by approximately 0.08 (0.61 → 0.53) and increases the standardized weight of quality (0.40 → 0.49), which remains the dominant modifiable predictor. The usage–intention zero-order correlation is r = 0.644.*
