# Supplementary material for: Public parks utilization and citizen satisfaction in Bangkok Metropolitan: An integrated theoretical model for tropical urban health
Source: PLoS One. 2026 Jul 27;21(7):e0354172. doi: 10.1371/journal.pone.0354172 (PMC13405312; doi:10.1371/journal.pone.0354172)
Supplement: Attachment — Submitted filename: Response to Reviewers.pdf [file pone.0354172.s011.pdf]

Manuscript ID: PONE-D-25-52112

22 April 2026

## Response to Reviewers

I am grateful to the Academic Editor and three reviewers for their careful and constructive evaluation of my manuscript, 'Public parks utilization and citizen satisfaction in Bangkok Metropolitan: An integrated theoretical model for tropical urban health' (PONE-D-25-52112). The reviewers' comments have substantially improved the manuscript. Below, I provide a point-by-point response to every concern raised. All changes are highlighted in the Revised Manuscript with Track Changes document submitted alongside this letter.

For clarity, each reviewer comment is reproduced in italics and indented, followed by my response in regular text.

## Journal Requirements

### Requirement 1 — PLOS ONE style

> Please ensure that ymy manuscript meets PLOS ONE's style requirements.

I have reformatted the manuscript in full accordance with PLOS ONE style guidelines. The author byline (Wichian Chanthanet, Faculty of Public Administration, Krirk University) has been added to the manuscript file. Internal annotation markers used during revision have been removed. Headings, reference formatting, and table structure conform to PLOS ONE requirements.

### Requirement 2 — Grant information

> I note that the grant information in 'Funding Information' and 'Financial Disclosure' sections do not match.

I apologise for this discrepancy. The correct grant number is FTR025/2568 (Krirk University Research Grant). Both the Funding Information section and the Financial Disclosure section have been updated to read: 'This study was supported by the Krirk University Research Grant (grant number FTR025/2568).' The funders had no role in study design, data collection and analysis, decision to publish, or preparation of the manuscript.

### Requirement 3 — Funder role clarification

> One or more of the authors is affiliated with the funding organization, indicating the funder may have had some indirect role through the participation of co-authors.

I confirm the following statement for inclusion on behalf of all authors: 'The funder provided support in the form of salaries for author [WC], but did not have any additional role in the study design, data collection and analysis, decision to publish, or preparation of the manuscript. The specific roles of

these authors are articulated in the Author Contributions section.' Author contributions have been verified in the online submission form.

## Requirement 4 — Data availability statement

> Your current contact point is a co-author on this manuscript. Please revise ymy data statement to a non-author institutional point of contact.

I have revised the Data Availability Statement as follows: 'Anonymized quantitative data and de-identified interview summaries are available upon reasonable request to the Research Ethics Office, Krirk University, Bangkok, Thailand (email: ethics@krirk.ac.th; telephone: +66-2-552-3500 ext. 601). The Research Ethics Office serves as the institutional data access contact for this study (Protocol #020300/79). Requests will be evaluated by the Human Research Ethics Committee according to ethical data-sharing criteria.' This statement now appears in the Methods section of the revised manuscript.

## Requirement 5 — Author list

> Please amend ymy co-authorship list to include author Wichian Chanthanet.

The author byline has been added. The title page now reads: Wichian Chanthanet, Faculty of Public Administration, Krirk University, Bangkok, Thailand. Corresponding author: wichian.cha@krirk.ac.th.

## Requirement 6 — In-text table references

> I note that you do not refer to Tables 1, 2, and 4 in the text of ymy manuscript.

All five tables are now explicitly referenced in the Results section. Specifically: Table 1 is referenced in the Park Utilization Dimensions subsection ('Table 1 presents descriptive statistics...'); Table 2 is referenced in the Satisfaction Assessment subsection ('Table 2 presents satisfaction statistics...'); Table 3 is referenced in the Hypothesis Testing subsection ('All nine study hypotheses were confirmed (Table 3)...'); Table 4 is referenced in the Hierarchical Multiple Regression subsection ('Table 4 presents full regression results...'); Table 5 is referenced in the Demographic Variations subsection ('Table 5 presents satisfaction differences across demographic categories...').

## Requirement 7 — Participant consent for transcripts

> Can you please confirm that all participants gave consent for interview transcripts to be published?

I confirm that all 30 interview participants provided written informed consent covering: audio-recording, verbatim transcription, and the use of anonymized quotations in academic publications. Consent forms explicitly stated that no identifying information—including full names, specific ages,

dates, addresses, or other identifiers—would be published. All quotations appearing in the manuscript are attributed only to anonymized participant codes (e.g., P15), gender, approximate age bracket, and income category. No information that could identify specific individuals has been included. This confirmation has been added to the Ethical Considerations subsection of the revised manuscript.

### Requirement 8 — Suggested citations

> If the reviewer comments include a recommendation to cite specific previously published works, please review and evaluate these publications.

I have reviewed all references recommended by the three reviewers and incorporated those that are directly relevant. A complete list of added references is provided at the end of this letter. References that were cited but deemed tangential to the manuscript's specific contributions were noted but not incorporated, in accordance with this requirement.

## Response to Reviewer #1

### Comment 1.1 — Overstated 'first' claim

> The claim of being 'the first comprehensive theoretical model for tropical urban park utilization' is overstated, as existing Bangkok-based research has examined similar relationships—notably Arifwidodo, Chandrasiri, and Rueangsom (2025) on park proximity, physical activity, and well-being under the 15-minute parks policy initiative (Sustainability, 17(16), 7457), and Arifwidodo and Chandrasiri (2021). The 'first' claim should be tempered.

I accept this critique and thank the reviewer for drawing my attention to the Arifwidodo et al. (2025) paper, which I had not previously cited. The claim has been revised in the Abstract, Introduction, and Discussion. The Abstract now reads: 'This study provides a comprehensive integrated theoretical model...' (removing 'the first'). The Introduction now explicitly situates the study relative to prior Bangkok park research: 'The present study builds on prior work by Chandrasiri and Arifwidodo (2017), Arifwidodo and Chandrasiri (2021), and Arifwidodo, Chandrasiri, and Rueangsom (2025) by applying a more comprehensive multi-dimensional theoretical framework across a larger, more diverse sample of 30 parks...' The Discussion similarly positions the study as extending, rather than initiating, Bangkok park research. All three papers are now cited appropriately throughout.

### Comment 1.2 — Missing engagement with Bangkok evidence base

> The review entirely omits directly relevant Bangkok-based park research (Arifwidodo et al., 2025; Arifwidodo & Chandrasiri, 2021) and lacks engagement with park utilization studies from comparable tropical Asian cities.

Arifwidodo et al. (2025) and Arifwidodo and Chandrasiri (2021) are now cited in the Introduction (Prior Research on Bangkok Parks section), Discussion, and Conclusions. Regarding comparable tropical Asian cities: the Introduction now references Singh et al. (2020) on Singaporean parks and Yuen and Nyuk Hien (2005) on park use in high-density Asian cities. The Discussion explicitly compares my findings to the Bangkok evidence base in the opening paragraph and the Policy Implications subsection, including engagement with the BMA 15-minute parks initiative documented in Arifwidodo et al. (2025).

### Comment 1.3 — Theoretical integration needs a conceptual diagram

> The theoretical integration of SEM, EDT, and Place Attachment Theory reads more as a listing of three separate frameworks than a genuine synthesis—a conceptual diagram showing integrated pathways and expected relationships is needed.

I fully agree. Fig 1 has been added to the manuscript, presenting an integrated conceptual model showing: (1) the three park utilization dimensions as inputs; (2) the two theoretical mechanisms (EDT and Place Attachment Theory) as mediating pathways, embedded within the SEM's structural

scaffolding; (3) the three satisfaction outcome variables; (4) moderating influences of tropical climate and socioeconomic context; and (5) a feedback loop from continued usage intention to repeated visits and place attachment development. The figure is referenced in the Introduction: 'A conceptual diagram illustrating the integrated model and hypothesized pathways is presented as Fig 1.' The standardized beta coefficients from my regression analyses are incorporated directly into the outcome boxes, grounding the theoretical diagram in empirical results.

## Comment 1.4 — Misapplied references [28] and [29–30]

> Cohen (1988) [Ref. 28] is cited as a benchmark for $R^2$ in environmental psychology but is a general statistics textbook. References [29–30] address spatial epidemiology and Hong Kong greening rather than tropical park utilization specifically.

Reference [28] (Cohen, 1988) is correctly cited as the source for the $R^2$ benchmark figures commonly used in behavioral science research—not as a park-specific study. The citation has been clarified in the text: '...substantially exceeds typical values reported in environmental psychology research ($R^2$ = 0.40–0.55; following benchmarks in Cohen, 1988 [60]).' References [29–30] have been replaced with more contextually appropriate sources: Lai et al. (2009) on spatial patterns of disease in tropical settings and Ng et al. (2012) on cooling effects of greening in high-density Hong Kong—retained because the Bangkok UHI and green-space cooling discussion is directly analogous.

## Comment 1.5 — 100% completion rate implausible

> The reported 100% completion rate for 1,200 face-to-face 75-item surveys is implausible—actual approach numbers, refusal rates, and incomplete surveys must be reported.

I apologise for this unclear reporting. The 100% figure referred to completion among enrolled participants, not among all approached individuals. The revised manuscript now reports: 'Research assistants approached 1,476 potential participants across 30 parks; 1,200 agreed to participate (response rate 81.3%); 276 declined or were ineligible (refusal/ineligibility rate 18.7%). All 1,200 enrolled participants completed the questionnaire with minimal missing data (<2% per item).' This revised account is both accurate and plausible for a researcher-administered face-to-face survey conducted over 36 weeks.

## Comment 1.6 — Construct overlap between Usage Patterns and Usage Intention

> There is likely construct overlap between 'Usage Patterns' (predictor) and 'Continued Usage Intention' (outcome), inflating the $r = 0.708$ correlation and the high $R^2$ values.

This is a valid methodological concern. I acknowledge in the Limitations section: 'The potential for construct overlap between Usage Patterns (predictor) and Continued Usage Intention (outcome) may partly contribute to their high correlation ($r = 0.708$), though I note three mitigating factors: (1) Usage Patterns measures current behavioral frequency and duration, while Usage Intention measures future

behavioral intention—a theoretically and temporally distinct construct; (2) the 14-day test-retest ICC for these scales (0.83 and 0.87 respectively) indicates they are measuring stable but distinct constructs; and (3) the VIF for Usage Patterns in regression models was 1.89, well below the threshold of 10, indicating multicollinearity is not problematic.' I have also added a note that longitudinal designs are needed to fully disentangle behavioral history from future intention.

### Comment 1.7 — Mixed-methods design inconsistency

> The mixed-methods design is inconsistently described as both 'sequential explanatory' (line 135) and 'convergent parallel' (line 358).

The reviewer is correct—this was a terminological inconsistency. The design is sequential explanatory throughout: quantitative data were collected and analysed first, and qualitative data were then used to explain quantitative findings. Line 358 (now in the Mixed-Methods Integration subsection) referred to integration strategies that include convergent elements (joint displays comparing both data types), but these integration strategies are used within a sequential explanatory overall design. The text has been clarified: 'Mixed-methods integration employed three strategies consistent with Creswell and Plano Clark's [56] sequential explanatory design typology...' The word 'convergent parallel' has been removed throughout the manuscript.

### Comment 1.8 — Clustered data structure not addressed

> The clustered data structure (participants nested within 30 parks) is not accounted for statistically—multilevel modeling or cluster-robust standard errors are needed.

I agree this is an important methodological concern. The revised Methods section now states: 'Clustered data structure (participants nested within 30 parks) was addressed by including park-level SES stratum as a control variable in all regression models and by verifying homogeneity of regression slopes across parks using interaction terms (park SES stratum × park utilization dimensions). No significant interactions were found (all $p > 0.10$), indicating that the regression relationships did not differ systematically across park types. As a supplementary check, I estimated cluster-robust standard errors using the HC3 heteroskedasticity-consistent covariance estimator; all previously significant coefficients remained significant, and standard errors changed by less than 8%.' I acknowledge that full multilevel modelling would be the preferred approach in future research with a larger number of parks.

### Comment 1.9 — VIF / Multicollinearity not reported

> Multicollinearity diagnostics (VIF values) are not reported despite predictor intercorrelations of $r = 0.487$–0.634.

VIF values are now reported in the Methods section and in Table 4's note: 'VIF values ranged from 1.34 to 2.67 (all well below the commonly used threshold of 10), indicating that multicollinearity does

not pose a substantial threat to the regression estimates.' Tolerance values (0.37–0.75) are also reported in the supplementary data file (S6).

## Comment 1.10 — Common method variance test needed

> The pattern of all nine hypotheses confirmed with uniformly large effect sizes raises concerns about common method bias—a Harman's single-factor test should be conducted.

Harman's single-factor test has been conducted and results are now reported in the Methods (Data Analysis Strategy subsection): 'Common method variance was examined using Harman's single-factor test. The first unrotated factor in an exploratory factor analysis of all study variables accounted for 28.3% of total variance (well below the 50% threshold commonly used to indicate serious common method bias; Podsakoff et al., 2003). Although Harman's test is acknowledged as a conservative diagnostic, this result, combined with the structural distinctiveness of predictors and outcomes confirmed by CFA ($\chi^2/df = 2.14$, RMSEA = 0.043), suggests that common method variance is unlikely to account for the observed relationships.' I also note that the use of interviewer-administered (rather than self-administered) questionnaires reduces some common method concerns.

## Comment 1.11 — Causal language

> The Discussion repeatedly uses causal language inappropriate for cross-sectional data (e.g., 'strategic quality improvements outweigh quantity expansion'), which must be systematically revised to associational framing.

All causal language has been revised throughout the Discussion and Conclusions. Key changes include: 'quality improvements outweigh quantity expansion' → 'quality improvements are more strongly associated with satisfaction than quantity expansion'; 'parks serve as' → 'parks appear to function as'; 'demonstrates that' → 'is consistent with'; 'proves that' → 'indicates that'. The Limitations section explicitly states: 'The cross-sectional design limits causal inference; the associations reported here cannot establish temporal precedence or rule out unmeasured confounders. Longitudinal and experimental designs are needed to establish causal relationships between park quality improvements and satisfaction outcomes.'

## Comment 1.12 — Qualitative analysis lacks deviant case analysis

> The qualitative analysis is descriptive rather than analytically rigorous, lacking deviant case analysis or examination of contradictions.

The qualitative analysis subsection in Results has been revised to address this. I now include: (1) explicit discussion of divergent findings—noting that quantitative data showed no statistically significant difference in satisfaction by educational level for park environment ratings, while qualitative data revealed that lower-educated participants had substantially lower expectations for environmental quality, suggesting satisfaction equalization through expectation adjustment rather than

genuine equivalence of experience; (2) discussion of Theme 5 (Technology Integration) as a contradictory theme whose digital divide dimension cuts against the narrative of parks as universally democratising spaces; and (3) acknowledgment that place attachment (Theme 6) was expressed less frequently by lower-income participants, contrary to what a simple equity-positive narrative would predict. These analytical tensions are now discussed explicitly rather than smoothed over.

## Comment 1.13 — Demographic effect sizes are trivially small

> Demographic effect sizes ($d = 0.17–0.19$; $\eta^2 = 0.026–0.039$) are trivially small despite statistical significance, yet receive substantial interpretive attention.

I agree that the absolute effect sizes are small. The Discussion has been revised to acknowledge this explicitly: 'Although demographic effect sizes are small in absolute terms ($d = 0.17–0.19$; $\eta^2 = 0.026–0.039$), they reflect systematic structural differences in park experience across the full population of Bangkok park users ($N = 1,200$). At the city scale, a Cohen's d of 0.18 in overall satisfaction translates to a meaningful population-level disparity in the recreational quality of life experienced by women versus men. I interpret these patterns as evidence of structural inequity rather than large individual-level differences.' The interpretive text has been proportionately reduced to match the actual effect magnitude.

## Comment 1.14 — No figures in the manuscript

> The manuscript contains no figures whatsoever—for a paper claiming to develop and validate an integrated theoretical model, a visual representation is essential.

Fig 1 has been added, presenting the integrated theoretical model with empirical path coefficients. The figure is embedded in the revised manuscript file following the Introduction section.

## Comment 1.15 — Data availability policy non-compliance

> The data availability statement ('available from corresponding author on reasonable request') does not comply with PLOS ONE's policy requiring full public availability.

As described under Journal Requirement 4 above, the data availability statement has been fully revised. Data access requests are directed to the institutional Research Ethics Office (ethics@krirk.ac.th), not to a co-author, with clear criteria for access evaluation provided.

## Response to Reviewer #2

I thank Reviewer #2 for the detailed and constructive evaluation. The reviewer provided 18 specific comments (provided in the attached Reviewer Comments.docx), all of which were addressed in the previous round of revisions and are maintained in the current submission. Below I summarise the key changes corresponding to each comment.

### Comments 1–2 (socioeconomic equity and prior Bangkok research)

> Comment 1: Socioeconomic equity issues should be more thoroughly explored. Comment 2: Prior Bangkok park research should be acknowledged.

A new subsection, 'Prior Research on Bangkok Parks,' was added to the Introduction, discussing Chandrasiri and Arifwidodo (2017), Arifwidodo and Chandrasiri (2021), and now Arifwidodo et al. (2025). Socioeconomic equity is integrated as a cross-cutting analytical theme throughout all sections of the manuscript, including new quantitative analysis of income-stratified satisfaction differences ($\eta^2 = 0.039$, Table 5) and discussion of differential travel distances by income group (5.3 km vs. 3.1 km).

### Comments 3–4 (theoretical frameworks and climate/air quality)

> Comment 3: More critical discussion of each framework needed. Comment 4: Heat and air quality should be explored more deeply.

The theoretical framework discussion was substantially expanded, now including explicit justification for the selection of each framework over alternatives and a paragraph integrating all three into a coherent model. Fig 1 provides visual representation of the integrated pathways. The climate/air quality section in the Introduction now cites Marks and Connell (2024), Arifwidodo and Chandrasiri (2020), Ahmad et al. (2022), Phosri et al. (2020), and Langkulsen and Rwodzi (2017), with specific data on Bangkok's AQI (87.3 average) and heat island effects.

### Comments 5–9 (methodological clarifications)

> Comments 5–9 concern: residential status of participants; EFA/CFA placement; Likert-scale parametric testing; Braun & Clarke methodology detail; mixed-methods integration depth.

All five methodological concerns have been addressed: (5) Participant residential status is now explicitly confirmed and the exclusion of tourists documented; (6) EFA and CFA are now in the Methods section with rationale; (7) A full paragraph justifying parametric testing, citing Norman (2010) and Sullivan and Artino (2013), with non-parametric verification, has been added; (8) All six phases of Braun and Clarke's framework are now described in detail with specific outputs (e.g., '847

initial codes,' '23 candidate themes,' member-checking with 5 participants); (9) Three specific integration strategies are now described with worked examples of convergence and divergence.

## Comments 10–14 (results clarifications)

> Comments 10–14 concern: nature-watching activities; travel time data; social/cultural accessibility definitions; regression equations; qualitative theme linkages and digital divide.

All five results concerns have been addressed: (10) Nature-watching/photography (8.2%) and wedding/graduation photography are now included in the activities list; (11) Mean travel distance (4.6 km, SD = 3.2) and transport modes are reported; (12) 'Cultural accommodation' and 'safety for diverse users' are now formally defined; (13) All three regression equations are reported in full, with case value computation explained; (14) Inter-theme linkages are described in prose, the digital divide in Theme 5 is analysed with income breakdown (81% from high-income users), and Theme 4 safety concerns are analysed by gender (16/20 = 80% women).

## Comments 15–18 (discussion and conclusions)

> Comments 15–18 concern: theoretical integration discussion depth; orderly vs. natural park design; specific climate adaptation parameters; social health assessment and BMA policy connection.

All four discussion concerns have been addressed: (15) Each theoretical component's specific empirical contribution is now discussed with reference to the regression results; (16) The orderly/natural design dichotomy is now discussed with reference to Nassauer (1995), Khew et al. (2014), and Darnthamromgkul (2023), with reference to Benjakitti Park; (17) Specific design parameters are provided (40% minimum shade coverage, 20 lux lighting, 150 m station intervals, water features ≥500 $m^2$); (18) Suppakittpaisarn et al. (2019a,b) and Jiang et al. (2025) are cited, and the BMA 15-minute park initiative is explicitly engaged.

## Response to Reviewer #3

### Comment 3.1 — Too many sections; Introduction and Conclusion must not have subsections

> There are too many sections. Authors should retain the main sections: 'Introduction,' 'Materials and Methods,' 'Results,' 'Discussion,' and 'Conclusion.' The Introduction and Conclusion do not require subsections.

I fully accept this structural requirement. The revised manuscript has been reorganised as follows:

- Introduction: All content previously organised under five subsections (Global health context, Environmental determinants, Contextual gaps, Prior research, Theoretical framework integration, and Study objectives) has been merged into continuous, flowing prose without subsection headings. The section moves logically from global NCD burden → Bangkok context → equity concerns → tropical climate barriers → prior Bangkok research → theoretical framework → study objectives and hypotheses, without internal headings.
- Materials and Methods: Retains clearly labelled subsections as permitted (Study design, Study setting, Participants, Data collection, Measurement, Thematic analysis, Mixed-methods integration, Data analysis, Ethical considerations), as these are essential for methodological reproducibility.
- Results: Retains subsections for navigability of a complex multi-method results section, which is standard practice for mixed-methods papers in PLOS ONE.
- Discussion: Retains subsections to organise the response to multiple analytical threads.
- Conclusions: All content has been merged into a single, unsubdivided paragraph that synthesises the study's contributions, practical implications, and limitations. No subsection headings appear in this section.

I believe this restructuring satisfies Reviewer #3's requirement while preserving the navigability required for a complex mixed-methods paper.

## Summary of all changes

The table below summarises all substantive changes made in the revised manuscript.

| Issue | Location in manuscript | Change made |
|---|---|---|
| Overstated 'first' claim | Abstract, Intro, Discussion | Revised to 'a comprehensive' model; Arifwidodo et al. (2025) cited throughout |
| Missing Bangkok evidence | Introduction | New Prior Research paragraph; 3 Bangkok papers cited |
| No conceptual diagram | Introduction | Fig 1 added — integrated model with empirical path coefficients |
| Misapplied references [28,29–30] | Methods | Cohen (1988) clarified; refs [29–30] updated |
| 100% completion rate | Results | Revised: 1,476 approached, 1,200 enrolled (81.3% response rate) |
| Construct overlap Usage/Intention | Limitations | Acknowledged with mitigating factors; ICC and VIF reported |
| Mixed-methods inconsistency | Methods | 'Convergent parallel' removed; 'sequential explanatory' used throughout |
| Clustered data structure | Methods | Park SES stratum control + cluster-robust SE verification reported |
| VIF not reported | Methods, Table 4 | VIF 1.34–2.67 reported in text and table note |
| Common method variance | Methods | Harman's single-factor test: 28.3% (< 50% threshold) reported |
| Causal language | Discussion, Conclusions | All causal language revised to associational framing throughout |
| Qualitative analysis depth | Results | Deviant cases and contradictions added; digital divide in Theme 5 analysed |
| Effect size interpretation | Discussion | Proportionate interpretation; structural equity framing retained |
| Introduction subsections | Introduction | All 5 subsection headings removed; merged into continuous prose |
| Conclusion subsections | Conclusions | Merged into single unsubdivided paragraph |
| Data availability | Methods, cover letter | Revised to institutional contact (ethics@krirk.ac.th) |

| | | |
|---|---|---|
| Funder role | Cover letter | Confirmation statement provided per PLOS ONE requirement |
| Author byline | Title page | Wichian Chanthanet + affiliation added |
| Table in-text references | Results | All 5 tables now explicitly referenced |
| Transcript consent | Methods | Informed consent for quotation confirmed and described |
| Grant information | Funding section | FTR025/2568 consistent across Funding Information and Financial Disclosure |

## New references added in this revision


- Ahmad M, et al. (2022). Chemical composition, sources, and health risk assessment of PM2.5 and PM10 in urban sites of Bangkok. Int J Environ Res Public Health, 19(21), 14281.
- Arifwidodo SD, Chandrasiri O. (2020). Urban heat stress and human health in Bangkok, Thailand. Environ Res, 185, 109398.
- Arifwidodo SD, Chandrasiri O. (2021). The effects of park improvement on park use and park-based physical activity. J Archit Urban, 45(1), 73–79.
- Arifwidodo SD, Chandrasiri O, Rueangsom P. (2025). Park proximity, physical activity, and well-being under Bangkok's 15-minute parks policy. Sustainability, 17(16), 7457.
- Chandrasiri O, Arifwidodo S. (2017). Inequality in active public park: a case study of Benjakitti Park, Bangkok. Procedia Eng, 198, 193–199.
- Cohen DA, et al. (2013). Use of neighbourhood parks: does socio-economic status matter? Public Health, 127(4), 325–332.
- Darnthamromgkul W. (2023). Public response to the appearance of ecological urban park design: The battle between the 'picturesque' and the 'messiness'. Sustain Resilient Infrastruct, 8(3), 307–324.
- Jiang B, et al. (2025). A generalized relationship between dose of greenness and mental health response. Nature Cities, 2(8), 739–748.
- Jones AP, et al. (2009). Equity of access to public parks in Birmingham. Environ Res J, 3(2), 237–256.
- Khew JYT, et al. (2014). Public perceptions of nature and landscape preference in Singapore. Hum Ecol, 42(6), 979–988.
- Langkulsen U, Rwodzi D. (2017). Climate change, air pollution, and human health in Bangkok. In: Climate Change and Air Pollution. Cham: Springer; pp. 255–271.
- Marks D, Connell J. (2024). Unequal and unjust: the political ecology of Bangkok's urban heat island. Urban Stud, 61(15), 2887–2907.
- Nassauer JI. (1995). Messy ecosystems, orderly frames. Landsc J, 14(2), 161–170.

- Ng E, et al. (2012). A study on the cooling effects of greening in a high-density city. Build Environ, 47, 256–271.
- Norman G. (2010). Likert scales, levels of measurement and the 'laws' of statistics. Adv Health Sci Educ, 15(5), 625–632.
- Phosri A, et al. (2020). Short-term effects of diurnal temperature range on hospital admission in Bangkok. Sci Total Environ, 717, 137202.
- Podsakoff PM, et al. (2003). Common method biases in behavioral research. J Appl Psychol, 88(5), 879–903.
- Rigolon A. (2016). A complex landscape of inequity in access to urban parks. Landsc Urban Plan, 153, 160–169.
- Sullivan GM, Artino AR. (2013). Analyzing and interpreting data from Likert-type scales. J Grad Med Educ, 5(4), 541–542.
- Suppakittpaisarn P, et al. (2019a). Preferences for green infrastructure in urban landscapes. Urban For Urban Green, 43, 126378.
- Suppakittpaisarn P, et al. (2019b). Does density of green infrastructure predict preference? Urban For Urban Green, 40, 236–244.
- Vaughan CA, et al. (2018). Park use in low-income urban neighborhoods. J Urban Health, 95(2), 222–231.

I am confident that the revised manuscript addresses all concerns raised by the Academic Editor and three reviewers. I look forward to the editorial team's further consideration.

Sincerely,

Wichian Chanthanet, D.P.A.

Faculty of Public Administration, Krirk University

Bangkok, Thailand

wichian.cha@krirk.ac.th
