# Supplementary material for: Public parks utilization and citizen satisfaction in Bangkok Metropolitan: An integrated theoretical model for tropical urban health
Source: PLoS One. 2026 Jul 27;21(7):e0354172. doi: 10.1371/journal.pone.0354172 (PMC13405312; doi:10.1371/journal.pone.0354172)
Supplement: Attachment — Submitted filename: Response_to_Reviewers_Corrected_Clean.pdf [file pone.0354172.s012.pdf]

Response to Reviewers

Manuscript PONE-D-25-52112R2

Public parks utilization and citizen satisfaction in Bangkok Metropolitan: An integrated theoretical model for tropical urban health

We thank the Editor and the three reviewers for their careful and constructive reading of the manuscript. The comments have substantially improved the paper. Below we respond to each point in turn; reviewer comments are shown in italics, followed by our response. All changes are visible in the tracked-changes version of the manuscript, and line references refer to that file.

Response to the Editor

Response: We have revised the manuscript to address all reviewer comments, with particular attention to (a) clarifying the contribution of research assistants given the single-author byline, (b) reframing externally derived design parameters so they are no longer presented as empirical findings, (c) adding a sensitivity analysis for the Usage Patterns / Usage Intention overlap, (d) supplying Figure 1 as a separate file per PLOS requirements, and (e) tempering interpretive language throughout the Results and Conclusions. A point-by-point response follows. We have also corrected minor inconsistencies noted during revision (e.g., an author–year citation in the Methods that should have been numbered [57], and the Thai-calendar year that should have read 2024).

Reviewer #1

1.1 Single authorship versus references to a research team and second coder

The Methods refer to "each researcher," to "two researchers" who independently coded 20% of transcripts (Cohen's κ = 0.83), and to conclusions developed "collaboratively by the research team," yet the manuscript lists a single author. This should be clarified.

Response: We have clarified this throughout the Methods. This is a single-author study: the coding framework, all analytic decisions, theme definition, interpretation, and writing were carried out by the author. The trained research assistants who supported field data collection (already noted in the manuscript) also performed independent second-coding of a 20% subsample for reliability assessment, working under the author's direct supervision and following the author's coding manual. This supporting technical role does not meet ICMJE authorship criteria. We have accordingly (i) replaced "each researcher" with "the author and a

trained research assistant" in Phase 1; (ii) replaced "Two researchers" with "The author and a trained research assistant" for the reliability coding; (iii) replaced "collaboratively by the research team" with "by the author, drawing on the coded outputs prepared with research-assistant support"; (iv) added an explicit statement of the single-author / supervised-assistant arrangement in the Qualitative thematic analysis subsection; and (v) added an Acknowledgments section recognizing the assistants and the expert panel. This single-author attribution is consistent with the project's grant documentation and final research report, which identify one principal investigator; the earlier plural wording was an editing inconsistency rather than an indication of an unlisted contributor.

1.2 Overlap between Usage Patterns and Continued Usage Intention (r = 0.644)

Given the strong correlation, a short supplementary analysis showing what happens to the Usage Intention model when Usage Patterns is dropped would reassure readers that the results are not an artifact of construct overlap.

Response: We have added a Supplementary sensitivity analysis paragraph to the regression Results and a new S1 Table. Removing Usage Patterns and re-estimating Continued Usage Intention on accessibility and quality yields standardized coefficients of 0.31 and 0.49, respectively, with $R^2 = 0.53$, compared with $R^2 = 0.61$ for the full three-predictor model (without demographic controls). Quality's standardized weight increases once the overlapping predictor is removed, so quality remains the dominant modifiable predictor and the central conclusion is unchanged. Removing Usage Patterns reduces explained variance by roughly 0.08 (0.61 → 0.53), confirming that the principal results are robust to the predictor–outcome overlap; the usage–intention zero-order correlation is $r = 0.644$. We also cross-reference this analysis in the Limitations.

1.3 Design parameters presented as findings

Specific values—40% shade coverage, 20 lux lighting, cooling stations every 150 m, water features ≥500 m²—read as empirical findings of this study, but they are external engineering/design targets. They should be reframed as drawn from the tropical-design literature.

Response: Agreed. In both the Discussion ("Tropical climate adaptations") and the Conclusions we now introduce these values explicitly as illustrative design targets drawn from established tropical urban design and microclimate literature [26], "rather than thresholds measured in the present study," and we state that they are "to be calibrated

locally." Hedging language ("on the order of," "roughly," "approximately") replaces the prescriptive minimum/maximum phrasing.

1.4 R^2 benchmark and its attribution

The claim that the model's R^2 substantially exceeds the 40–55% typical of environmental psychology is attributed to a general statistics source (Cohen, 1992 [42]) and reads as a claim of model superiority. Either a more appropriate citation or softer framing is needed.

Response: We have softened the framing in the Abstract, Discussion, and Conclusions. The Abstract no longer claims the model exceeds typical research; it now notes the relatively high R^2 "partly reflects the common-method effects inherent in self-report designs." The Discussion now reads that the R^2 "is high relative to typical R^2 values in behavioral cross-sectional research" and should be "interpreted cautiously" given common-method variance and predictor–outcome proximity, "rather than indicating inherent model superiority." Reference [42] is retained only as a general benchmark for typical effect sizes, consistent with your suggestion to either re-cite or soften.

1.5 Citation of Lai et al. for climate constraints

Lai et al. (a disease-mapping text) is cited to support claims about tropical climate constraints on outdoor activity; Phosri et al. or Marks and Connell would fit better.

Response: We have replaced the in-text citation for the climate-constraint claim in the Introduction, which now cites Phosri et al. [60] together with Ng et al. [26]. Lai et al. [25] is retained in the reference list and is now cited where it is genuinely apposite—in the Discussion, supporting the use of spatial-epidemiological approaches to target environmental health interventions to high-AQI zones. Reference numbering is therefore unchanged.

1.6 Response-letter typographical errors

The previous response letter contained typographical errors (e.g., "ymy").

Response: This letter has been proofread carefully to remove such errors.

Reviewer #2

2.1 Thai calendar year for the AQI figure

On p. 43 the AQI value is dated "2567" (Thai Buddhist calendar). The international year should be used.

Response: Corrected. The Introduction now reads "the first six months of 2024."

2.2 Figure 1 not visible in the PDF

Figure 1 does not appear in the compiled PDF.

Response: We regret the earlier rendering problem, which arose because the figure had not been provided as a separate figure file for the submission system to assemble into the review PDF. In this revision we follow PLOS figure requirements: Figure 1 is supplied as a separate file (Fig1.tif, 300 dpi, within PLOS size limits) and is no longer embedded in the manuscript file, while the figure caption remains in the manuscript text immediately after the paragraph in which Fig 1 is first cited. The submission system will therefore assemble the figure into the reviewer PDF automatically, resolving the reviewer's concern.

2.3 Sample skews toward higher income and education

Participants' incomes and education levels are relatively high; the implications for generalizability should be discussed.

Response: We have expanded the Limitations to address this directly. We note that the sample is more educated and higher-income than the wider Bangkok population (50.3% with at least a bachelor's degree; ~33% with household income above 35,000 THB/month), that on-site recruitment of current users is the most likely explanation, and that this skew implies our reported satisfaction levels may be conservative for the most disadvantaged residents and that the documented socioeconomic gradients likely understate true population-level inequity. This strengthens rather than weakens the equity argument.

2.4 Correlation among demographic independent variables

The demographic predictors entered in the regressions are likely correlated; the implications should be addressed.

Response: We have added text to the Data analysis strategy noting that education and income in particular are inter-correlated, reporting that variance inflation factors for the full predictor set ranged from 1.00 to 2.06 (Table 4)—well below conventional thresholds—and advising that the small demographic coefficients be read as adjusted associations net of shared variance rather than as independent effects. The demographic block as a whole explains only 2.8–3.4% of variance (Step 1).

Reviewer #3 and Editor

3.1 Subjective statements beyond the evidence in Results and Conclusions

Several statements in the Results and Conclusions are interpretive and go beyond what the cross-sectional evidence supports; these should be tempered.

Response: We have tempered interpretive language throughout. Examples: in the Results, "reflecting the fundamental mismatch" is now "consistent with a mismatch"; in the Discussion, "directly challenging individual behavior-change interventions" is now "suggesting that individual behavior-change interventions … may be insufficient," and "the most significant and actionable finding" is now "one of the most policy-relevant findings"; in the Abstract and Conclusions, "demonstrating/demonstrates" is now "indicating/indicates," and "several significant contributions" is now "several contributions." Causal phrasing has been replaced with associational phrasing wherever the design does not support causal inference.

Summary of changes

The principal revisions are: (1) clarified single-author/RA-supervision arrangement plus a new Acknowledgments section; (2) a new sensitivity analysis (S1 Table) for the Usage Patterns / Usage Intention overlap; (3) reframing of design parameters as literature-derived illustrative targets; (4) softened R^2 framing in the Abstract, Discussion, and Conclusions; (5) corrected and re-homed citations (Phosri [60]/Ng [26] for climate constraints; Lai [25] re-cited appropriately; Marks & Connell numbered [57]); (6) "2024" replacing "2567"; (7) Figure 1 removed from the manuscript file and supplied as a separate Fig1.tif (caption retained in text) per PLOS requirements; (8) expanded Limitations on sample representativeness and demographic inter-correlation; and (9) tempered interpretive language throughout. We believe these changes address all reviewer concerns and we thank the reviewers again for their helpful guidance.
