# Supplementary material for: Public parks utilization and citizen satisfaction in Bangkok Metropolitan: An integrated theoretical model for tropical urban health
Source: PLoS One. 2026 Jul 27;21(7):e0354172. doi: 10.1371/journal.pone.0354172 (PMC13405312; doi:10.1371/journal.pone.0354172)
Supplement: Attachment — Submitted filename: Reviewer Comments.docx [file pone.0354172.s009.docx]

**Reviewer Comments for *Public parks utilization and citizen satisfaction in Bangkok Metropolitan: An integrated theoretical model for tropical urban health***

This manuscript takes an interesting data collection and analytical approach to investigate interactions between demographics, park design, and user experience to make recommendations with respect to improving community resilience to climate change and enhancing community health and wellbeing in a tropical climate. Potentially, this manuscript could make a very important contribution to the literature, but before I could recommend publication, the authors would need to adequately address some of my concerns. My greatest concern, in general, is that the paper reads more like a report than an academic manuscript, with too many abbreviated discussions and bullet points. The following specific comments address this general observation, as well as including some additional points:

1. P. 10, L 62-64 - *Unlike private fitness facilities or sports clubs that may exclude lower-income populations, public parks offer democratic access to health-promoting environments regardless of socioeconomic status.* This is an important point and one much discussed in the literature, but such discussions are not explored here. See, for example, Jones et al., 2009; Cohen et al., 2013; Rigolon, 2016; Vaughan et al., 2018. Many argue that the co-benefits provided by Nature-based Solutions, including community well-being, must be equitably distributed in order to meet the true spirit of the concept. These socio-economic and gender issues should be more thoroughly explored throughout the manuscript.

Cohen, D. A., Lapham, S., Evenson, K. R., Williamson, S., Golinelli, D., Ward, P., ... & McKenzie, T. L. (2013). Use of neighbourhood parks: does socio-economic status matter? A four-city study. *Public health*, *127*(4), 325-332.

Jones, A. P., Brainard, J., Bateman, I. J., & Lovett, A. A. (2009). Equity of access to public parks in Birmingham, England. *Environmental Research Journal*, *3*(2), 237-256.

Rigolon, A. (2016). A complex landscape of inequity in access to urban parks: A literature review. *Landscape and urban planning*, *153*, 160-169.

Vaughan, C. A., Colabianchi, N., Hunter, G. P., Beckman, R., & Dubowitz, T. (2018). Park use in low-income urban neighborhoods: who uses the parks and why?. *Journal of urban health*, *95*(2), 222-231.

2. It is noted in the Introduction, and elsewhere, that there is a gap in the understanding of park usage and design for tropical regions. I would note, however, that research on user experience and perspectives had been done for the different phases of Benjakitti Park in Bangkok (Chandrasiri and Arifwidodo, 2017; Arifwidodo and Chandrasiri, 2021).

Arifwidodo, S. D., & Chandrasiri, O. (2021). The effects of park improvement on park use and park-based physical activity. *Journal of Architecture and Urbanism*, *45*(1), 73-79.

Chandrasiri, O., & Arifwidodo, S. (2017). Inequality in active public park: a case study of Benjakitti Park in Bangkok, Thailand. *Procedia engineering*, *198*, 193-199.

3. P. 11 – the theoretical frameworks employed in the study and the idea of their integration is interesting, but a more detailed, critical, assessment of each framework is needed. Why were these frameworks chosen over other, alternative frameworks?
4. P. 12-13 – there is no doubt that heat and air quality can impact individual health and activities in Bangkok, but these issues should be explored more deeply (e.g. Langkulsen and Rwodzi, 2017; Arifwidodo and Chandrasiri, 2020; Phosri et al., 2020; Ahmad et al., 2022; Marks and Connell, 2024).

Ahmad, M., Manjantrarat, T., Rattanawongsa, W., Muensri, P., Saenmuangchin, R., Klamchuen, A., ... & Panyametheekul, S. (2022). Chemical composition, sources, and health risk assessment of PM2. 5 and PM10 in urban sites of Bangkok, Thailand. *International journal of environmental research and public health*, *19*(21), 14281.

Arifwidodo, S. D., & Chandrasiri, O. (2020). Urban heat stress and human health in Bangkok, Thailand. *Environmental research*, *185*, 109398.

Langkulsen, U., & Rwodzi, D. (2017). Climate change, air pollution, and human health in Bangkok. In *Climate Change and Air Pollution: The Impact on Human Health in Developed and Developing Countries* (pp. 255-271). Cham: Springer International Publishing.

Marks, D., & Connell, J. (2024). Unequal and unjust: The political ecology of Bangkok’s increasing urban heat island. *Urban Studies*, *61*(15), 2887-2907.

Phosri, A., Sihabut, T., & Jaikanlaya, C. (2020). Short-term effects of diurnal temperature range on hospital admission in Bangkok, Thailand. *Science of the Total environment*, *717*, 137202.

**Methods**

5. P. 13 – for some, but not all, public parks in Bangkok, a sizable proportion of users will be international tourists in addition to local people. Potentially, this pool of users will have different reflections on park amenities, design, etc. than local people, or the international community living and working on a longer-term basis in Thailand. Did you identify the users according to the location of their primary residence?
6. P. 17 – Why are the CFA and EFA results in the Methodology section? Furthermore, the discussion of CFA and EFA theory related to this type of study is quite short on detail. Please discuss in greater depth the specifics of the methodology for each analytical approach.
7. The quantitative analysis was done using Likert scale data, but there is some debate on whether it is appropriate to use parametric statistical testing with such quasi-quantitative scaling/ranking data. This discussion should be acknowledged and support for using parametric testing should be included.
8. The Braun and Clarke method of thematic analysis is appropriate and well-established for this type of study, but little detail is provided in the paper on the analytical methodology. Deeper discussion should be provided, as currently this section reads more like a report.
9. Mixed-methods integration – this is an important aspect of the study methodology, but it is summarized using 3 bullet points and supported by one reference. A deeper, critical discussion regarding the integration is warranted.

**Results**

10. P. 20, L. 420-428 - Park user activities – no one used the parks for nature-watching/photography? Other photo opportunities, including graduations and weddings?
11. Did you identify travel times for people to reach the park from home? What were the modes of transportation usually employed to reach the park?
12. Table 1 – how do you define social accessibility and cultural accommodation? The key observations for Table 1 are simply a list of bullet points. Deeper discussion of each bullet point is warranted.
13. It might be helpful to show the actual multiple regression equations for clarity of interpretation. In the multiple regression analysis please explicitly explain how you determined the case values for accessibility, quality, and usage patterns.
14. Braun and Clarke thematic analysis results – often the themes and sub-themes identified from surveys are shown graphically, where the themes and sub-themes are explicitly linked. In this paper, themes are presented independently, but the linkages between themes also can provide revealing interpretation of system patterns. Theme 5, Technology integration expectations – the qualitative examples all are from high-income users and this suggests a digital divide. Yet, smart phone use, online banking, QR purchases are common throughout the Thai demographics. This theme requires a deeper exploration. There is some suggestion that the themes may have gendered nuances (e.g. safety concerns), but this issue warrants a deeper analysis.

**Discussion**

15. P. 28 – the material on the Theoretical Integration Success is very interesting and important, but the discussion of each theory consists of only one sentence, again, more like a report. A richer discussion here is warranted and really will be an important contribution.
16. P. 29 – Park quality, especially environmental quality enhancement, such as landscaping maintenance, may be quite difficult to define. Singapore, for example, while being a renowned biophilic city, generally prefers orderly, highly maintained greenspace (e.g. Khew et al., 2014) as compared to a “messy” natural presentation. This design dichotomy of orderly vs messy (natural) has been examined in North America and Thailand (Nassauer, 1995; Darnthamromgkul, 2023). Certainly, this contrast is visible in Bangkok’s Benjakitti Park between the older, grid park and the newer, natural, forest park.

Darnthamromgkul, W. (2023). Public response to the appearance of ecological urban park design: The battle between the ‘picturesque’ and the ‘messiness’. *Sustainable and Resilient Infrastructure*, *8*(3), 307-324.

Khew, J. Y. T., Yokohari, M., & Tanaka, T. (2014). Public perceptions of nature and landscape preference in Singapore. *Human Ecology*, *42*(6), 979-988.

Nassauer, J. I. (1995). Messy ecosystems, orderly frames. *Landscape journal*, *14*(2), 161-170.

17. P. 29 – Essential climate adaptations - *Extended usage opportunities: Enhanced evening lighting, covered activity areas, climate-controlled indoor spaces* – I should imagine that enhanced evening lighting would not only be attractive to tropical climates, but temperate climates as well. Overall, while the essential climate adaptation recommendations are reasonable, it would be helpful to have more detail. For example, under *Microclimate management: Strategic landscaping, water features, vegetation barriers, wind corridors* – what is meant by strategic landscaping? Can you provide examples? How big do water features need to be to effectively cool?
18. P. 29 - *Policy implications for social health:* As flagged in my point 17, while the recommendations made here are reasonable, more detail and a deeper discussion would be helpful. How is social health assessed in relation to green infrastructure? You could see, for example, Suppakittpaisarn et al., 2019a,b; and Jiang et al. 2025. Also, how do these recommendations tie in with the existing BMA policies of 15-minute parks and other greening policies for the city?

Jiang, B., Li, J., Gong, P., Webster, C., Schumann, G., Liu, X., & Suppakittpaisarn, P. (2025). A generalized relationship between dose of greenness and mental health response. *Nature Cities*, *2*(8), 739-748.

Suppakittpaisarn, P., Larsen, L., & Sullivan, W. C. (2019a). Preferences for green infrastructure and green stormwater infrastructure in urban landscapes: Differences between designers and laypeople. *Urban Forestry & Urban Greening*, *43*, 126378.

Suppakittpaisarn, P., Jiang, B., Slavenas, M., & Sullivan, W. C. (2019b). Does density of green infrastructure predict preference?. *Urban forestry & urban greening*, *40*, 236-244.

**Conclusions**

Many recommendations are made, most of which are reasonable, but details on exact configuration and implementation are limited. For example, *Climate-specific Insights: Identification of fundamental design requirements for tropical urban parks that differ markedly from temperate climate guidelines, including essential heat protection systems and extended evening usage opportunities.* But what exactly are these design requirements? Here, you would need to lean on details from landscape architecture and urban design with respect to thermal comfort. Is canopy cover/density most important? A mix of vegetation types? Water features? The size of water features? Park size? Park shape? Park distribution within the overall urban fabric mix? Is park connectivity important? Furthermore, in Environmental Improvements, much already is being done with IoT and big data for environmental monitoring. For example, the IQAir network (<https://www.iqair.com/air-quality-map/thailand/bangkok/bangkok>) of air quality sensors has around 450 stations reporting PM2.5 for the city.

**Supporting Information**

S5 only seems to have the interview questions and does not include the thematic analysis procedures (p. 37, L. 975).
