# Supplementary material for: Public parks utilization and citizen satisfaction in Bangkok Metropolitan: An integrated theoretical model for tropical urban health
Source: PLoS One. 2026 Jul 27;21(7):e0354172. doi: 10.1371/journal.pone.0354172 (PMC13405312; doi:10.1371/journal.pone.0354172)
Supplement: Peer review history

# Peer review: Decision Letter 0

**DOI:** 10.1371/journal.pone.0354172.r001

**Authors:** Weicong Li

16 Apr 2026

PONE-D-25-52112

Public parks utilization and citizen satisfaction in Bangkok Metropolitan: An integrated theoretical model for tropical urban health

PLOS One

Dear Dr. Chanthanet,

Thank you for submitting your manuscript to PLOS ONE. After careful consideration, we feel that it has merit but does not fully meet PLOS ONE’s publication criteria as it currently stands. Therefore, we invite you to submit a revised version of the manuscript that addresses the points raised during the review process.

Please submit your revised manuscript by May 31 2026 11:59PM. If you will need more time than this to complete your revisions, please reply to this message or contact the journal office at plosone@plos.org. When you're ready to submit your revision, log on to https://www.editorialmanager.com/pone/ and select the 'Submissions Needing Revision' folder to locate your manuscript file.

Please include the following items when submitting your revised manuscript:

- A letter that responds to each point raised by the academic editor and reviewer(s). You should upload this letter as a separate file labeled 'Response to Reviewers'.
- A marked-up copy of your manuscript that highlights changes made to the original version. You should upload this as a separate file labeled 'Revised Manuscript with Track Changes'.
- An unmarked version of your revised paper without tracked changes. You should upload this as a separate file labeled 'Manuscript'.

If you would like to make changes to your financial disclosure, please include your updated statement in your cover letter. Guidelines for resubmitting your figure files are available below the reviewer comments at the end of this letter.

If applicable, we recommend that you deposit your laboratory protocols in protocols.io to enhance the reproducibility of your results. Protocols.io assigns your protocol its own identifier (DOI) so that it can be cited independently in the future. For instructions see: https://journals.plos.org/plosone/s/submission-guidelines#loc-laboratory-protocols. Additionally, PLOS ONE offers an option for publishing peer-reviewed Lab Protocol articles, which describe protocols hosted on protocols.io. Read more information on sharing protocols at https://plos.org/protocols?utm_medium=editorial-email&utm_source=authorletters&utm_campaign=protocols.

As the corresponding author, your ORCID iD is verified in the submission system and will appear in the published article. PLOS supports the use of ORCID, and we encourage all coauthors to register for an ORCID iD and use it as well. Please encourage your coauthors to verify their ORCID iD within the submission system before final acceptance, as unverified ORCID iDs will not appear in the published article. *Only*  the individual author can complete the verification step; PLOS staff *cannot*  verify ORCID iDs on behalf of authors.

We look forward to receiving your revised manuscript.

Kind regards,

Weicong Li, P.hD

Academic Editor

PLOS One

Journal Requirements:

When submitting your revision, we need you to address these additional requirements.

1. Please ensure that your manuscript meets PLOS ONE's style requirements, including those for file naming. The PLOS ONE style templates can be found at

https://journals.plos.org/plosone/s/file?id=wjVg/PLOSOne_formatting_sample_main_body.pdf and

https://journals.plos.org/plosone/s/file?id=ba62/PLOSOne_formatting_sample_title_authors_affiliations.pdf

2. We note that the grant information you provided in the ‘Funding Information’ and ‘Financial Disclosure’ sections do not match.

When you resubmit, please ensure that you provide the correct grant numbers for the awards you received for your study in the ‘Funding Information’ section.

3. Thank you for stating the following financial disclosure:

“The author received specific funding for this work from the Krirk University Research Grant (FTR025/2568). The funders had no role in study design, data collection and analysis, the decision to publish, or the preparation of the manuscript.”

We note that one or more of the authors is affiliated with the funding organization, indicating the funder may have had some role in the design, data collection, analysis or preparation of your manuscript for publication; in other words, the funder played an indirect role through the participation of the co-authors. If the funding organization did not play a role in the study design, data collection and analysis, decision to publish, or preparation of the manuscript and only provided financial support in the form of authors' salaries and/or research materials, please do the following:

1. Review your statements relating to the author contributions, and ensure you have specifically and accurately indicated the role(s) that these authors had in your study. These amendments should be made in the online form.

2. Confirm in your cover letter that you agree with the following statement, and we will change the online submission form on your behalf:

“The funder provided support in the form of salaries for authors [insert relevant initials], but did not have any additional role in the study design, data collection and analysis, decision to publish, or preparation of the manuscript. The specific roles of these authors are articulated in the ‘author contributions’ section.

4. In the online submission form you indicate that your data is not available for proprietary reasons and have provided a contact point for accessing this data. Please note that your current contact point is a co-author on this manuscript. According to our Data Policy, the contact point must not be an author on the manuscript and must be an institutional contact, ideally not an individual. Please revise your data statement to a non-author institutional point of contact, such as a data access or ethics committee, and send this to us via return email. Please also include contact information for the third party organization, and please include the full citation of where the data can be found.

5. Please amend your authorship list in your manuscript file to include author Wichian Chanthanet.

6. We note you have included a table to which you do not refer in the text of your manuscript. Please ensure that you refer to Tables 1, 2 and 4 in your text; if accepted, production will need this reference to link the reader to the Table.

7. We note that this data set consists of interview transcripts. Can you please confirm that all participants gave consent for interview transcript to be published?

If they DID provide consent for these transcripts to be published, please also confirm that the transcripts do not contain any potentially identifying information (or let us know if the participants consented to having their personal details published and made publicly available). We consider the following details to be identifying information:

- Names, nicknames, and initials

- Age more specific than round numbers

- GPS coordinates, physical addresses, IP addresses, email addresses

- Information in small sample sizes (e.g. 40 students from X class in X year at X university)

- Specific dates (e.g. visit dates, interview dates)

- ID numbers

Or, if the participants DID NOT provide consent for these transcripts to be published:

- Provide a de-identified version of the data or excerpts of interview responses

- Provide information regarding how these transcripts can be accessed by researchers who meet the criteria for access to confidential data, including:

a) the grounds for restriction

b) the name of the ethics committee, Institutional Review Board, or third-party organization that is imposing sharing restrictions on the data

c) a non-author, institutional point of contact that is able to field data access queries, in the interest of maintaining long-term data accessibility.

d) Any relevant data set names, URLs, DOIs, etc. that an independent researcher would need in order to request your minimal data set.

For further information on sharing data that contains sensitive participant information, please see: https://journals.plos.org/plosone/s/data-availability#loc-human-research-participant-data-and-other-sensitive-data

If there are ethical, legal, or third-party restrictions upon your dataset, you must provide all of the following details (https://journals.plos.org/plosone/s/data-availability#loc-acceptable-data-access-restrictions):

1. A complete description of the dataset

2. The nature of the restrictions upon the data (ethical, legal, or owned by a third party) and the reasoning behind them

3. The full name of the body imposing the restrictions upon your dataset (ethics committee, institution, data access committee, etc)

4. If the data are owned by a third party, confirmation of whether the authors received any special privileges in accessing the data that other researchers would not have

5. Direct, non-author contact information (preferably email) for the body imposing the restrictions upon the data, to which data access requests can be sent

8. If the reviewer comments include a recommendation to cite specific previously published works, please review and evaluate these publications to determine whether they are relevant and should be cited. There is no requirement to cite these works unless the editor has indicated otherwise.

Additional Editor Comments:

Please find my comment in reviewer 3. Authors must revise their suggestions and respond to each reviewer's comments.

[Note: HTML markup is below. Please do not edit.]

Reviewers' comments:

Reviewer's Responses to Questions

**Comments to the Author**

1. Is the manuscript technically sound, and do the data support the conclusions?

The manuscript must describe a technically sound piece of scientific research with data that supports the conclusions. Experiments must have been conducted rigorously, with appropriate controls, replication, and sample sizes. The conclusions must be drawn appropriately based on the data presented.

Reviewer #1: Partly

Reviewer #2: Partly

Reviewer #3: Partly

**********

2. Has the statistical analysis been performed appropriately and rigorously?

Reviewer #1: Yes

Reviewer #2: No

Reviewer #3: N/A

**********

3. Have the authors made all data underlying the findings in their manuscript fully available?

The PLOS Data policy requires authors to make all data underlying the findings described in their manuscript fully available without restriction, with rare exception (please refer to the Data Availability Statement in the manuscript PDF file). The data should be provided as part of the manuscript or its supporting information, or deposited to a public repository. For example, in addition to summary statistics, the data points behind means, medians and variance measures should be available. If there are restrictions on publicly sharing data—e.g. participant privacy or use of data from a third party—those must be specified.

Reviewer #1: Yes

Reviewer #2: Yes

Reviewer #3: Yes

**********

4. Is the manuscript presented in an intelligible fashion and written in standard English?

PLOS ONE does not copyedit accepted manuscripts, so the language in submitted articles must be clear, correct, and unambiguous. Any typographical or grammatical errors should be corrected at revision, so please note any specific errors here.

Reviewer #1: Yes

Reviewer #2: Yes

Reviewer #3: Yes

**********

5. Review Comments to the Author

Please use the space provided to explain your answers to the questions above. You may also include additional comments for the author, including concerns about dual publication, research ethics, or publication ethics. (Please upload your review as an attachment if it exceeds 20,000 characters)

Reviewer #1: The study addresses a legitimate gap regarding park utilization in tropical megacities, and Bangkok provides an excellent setting given its NCD burden and limited green space. The ambition to integrate three theoretical frameworks is noteworthy. However, the claim of being "the first comprehensive theoretical model for tropical urban park utilization" is overstated, as existing Bangkok-based research has examined similar relationships — notably Arifwidodo, Chandrasiri, and Rueangsom (2025) on park proximity, physical activity, and well-being under the 15-minute parks policy initiative (Sustainability, 17(16), 7457), and Arifwidodo and Chandrasiri (2021) on park improvement effects on park use and park-based physical activity (Journal of Architecture and Urbanism, 45(1), 73–79). The "first" claim should be tempered, and the practical policy implications would be substantially strengthened by engaging with this existing Bangkok evidence base rather than positioning the study in isolation.

The introduction competently overviews the global NCD burden and parks-health relationship, but contains critical gaps. Most significantly, the review entirely omits directly relevant Bangkok-based park research (Arifwidodo et al., 2025; Arifwidodo & Chandrasiri, 2021) and lacks engagement with park utilization studies from comparable tropical Asian cities such as Singapore, Kuala Lumpur, or Jakarta. The theoretical integration of SEM, EDT, and Place Attachment Theory reads more as a listing of three separate frameworks than a genuine synthesis — a conceptual diagram showing integrated pathways and expected relationships is needed. Additionally, some references are misapplied: Cohen (1988) [Ref. 24] is cited as a benchmark for R² in environmental psychology but is a general statistics textbook, while references [25–26] address spatial epidemiology and Hong Kong greening rather than tropical park utilization specifically.

Several significant methodological concerns require attention. The reported 100% completion rate for 1,200 face-to-face 75-item surveys is implausible — actual approach numbers, refusal rates, and incomplete surveys must be reported. Sampling only current park users fundamentally constrains the policy conclusions drawn about "universal access" and "health equity," as non-user barriers are entirely unrepresented. There is likely construct overlap between "Usage Patterns" (predictor) and "Continued Usage Intention" (outcome), inflating the r = 0.708 correlation and the high R² values. The mixed-methods design is inconsistently described as both "sequential explanatory" (line 135) and "convergent parallel" (line 358), which are fundamentally different designs. The clustered data structure (participants nested within 30 parks) is not accounted for statistically — multilevel modeling or cluster-robust standard errors are needed. Finally, multicollinearity diagnostics (VIF values) are not reported despite predictor intercorrelations of r = 0.487–0.634.

The pattern of all nine hypotheses confirmed with uniformly large effect sizes and R² values of 0.60–0.66 raises concerns about construct overlap, common method bias (all self-report measures from the same respondents at the same time), or selective reporting — a common method variance test (e.g., Harman's single-factor test) should be conducted and reported. The Discussion repeatedly uses causal language inappropriate for cross-sectional data (e.g., "strategic quality improvements outweigh quantity expansion"), which must be systematically revised to associational framing. The qualitative analysis is descriptive rather than analytically rigorous, lacking deviant case analysis or examination of contradictions, reading more as confirmation of quantitative findings than an independent contribution. Demographic effect sizes (d = 0.17–0.19; η² = 0.026–0.039) are trivially small despite statistical significance, yet receive substantial interpretive attention. Critically, the Discussion does not engage with existing Bangkok-based park evidence, particularly Arifwidodo et al. (2025) and Arifwidodo and Chandrasiri (2021), which would directly contextualize the "quality-first" argument.

The manuscript is generally well-organized and clearly written, but at approximately 9,000 words it is excessively long for PLOS ONE, with the Conclusions section reading more like a planning guideline than a research paper conclusion. Most critically, the manuscript contains no figures whatsoever — for a paper claiming to develop and validate an integrated theoretical model, a visual representation of that model is essential. The "Key Observations" and "Key Findings" bullet points after Tables 1–2 are redundant with the narrative text and should be removed. Finally, the data availability statement ("available from corresponding author on reasonable request") does not comply with PLOS ONE's policy requiring full public availability without restriction; the authors must deposit anonymized data in a public repository or provide an acceptable justification.

Reviewer #2: Generally an interesting paper that has the potential to be an important contribution to the literature. However, the manuscript reads more like a report than a scientific paper. Deeper discussion of key methods, results, and recommendations are needed

Please see attached file and I hope my comments can help to strengthen your paper.

Reviewer #3: One important issue is that there are too many sections. Authors should retain the main sections: “Introduction,” “Materials and Methods,” “Results,” “Discussion,” and “Conclusion.” Each of these sections may then include a few subsections, but the number should not be excessive. The Introduction and Conclusion do not require subsections.

**********

6. PLOS authors have the option to publish the peer review history of their article (what does this mean?). If published, this will include your full peer review and any attached files.

If you choose “no”, your identity will remain anonymous but your review may still be made public.

**Do you want your identity to be public for this peer review?**  For information about this choice, including consent withdrawal, please see our Privacy Policy.

Reviewer #1: No

Reviewer #2: No

Reviewer #3: No

**********

[NOTE: If reviewer comments were submitted as an attachment file, they will be attached to this email and accessible via the submission site. Please log into your account, locate the manuscript record, and check for the action link "View Attachments". If this link does not appear, there are no attachment files.]

To ensure your figures meet our technical requirements, please review our figure guidelines: https://journals.plos.org/plosone/s/figures

You may also use PLOS’s free figure tool, NAAS, to help you prepare publication quality figures: https://journals.plos.org/plosone/s/figures#loc-tools-for-figure-preparation.

NAAS will assess whether your figures meet our technical requirements by comparing each figure against our figure specifications.

Attachment Submitted filename: Reviewer Comments.docx

# Author comment: Author response to Decision Letter 1

**DOI:** 10.1371/journal.pone.0354172.r002

25 Apr 2026

Thank you for the opportunity to revise and resubmit my manuscript (Manuscript ID: PONE-D-25-52112).

In response to the editorial and reviewer comments, I have carefully revised the manuscript and addressed all concerns raised. I have provided a detailed, point-by-point response to each comment in the "Response to Reviewers" document. All corresponding changes have been clearly indicated in the revised manuscript with track changes.

Key revisions include the following:

Full compliance with journal formatting and style requirements

Clarification of funding information and author details

Revision of the Data Availability Statement to an institutional, non-author contact point

Addition of a conceptual figure illustrating the integrated theoretical model

Strengthening of the methodological and analytical sections, including reporting of response rates, multicollinearity diagnostics, and common method bias assessment

Refinement of theoretical positioning and engagement with relevant literature

Revision of language to ensure appropriate interpretation of cross-sectional findings

I believe these revisions have substantially improved the clarity, rigor, and contribution of the manuscript.

Thank you for your time and consideration. I look forward to your feedback.

Attachment Submitted filename: Response to Reviewers.pdf

# Peer review: Decision Letter 1

**DOI:** 10.1371/journal.pone.0354172.r003

**Authors:** Weicong Li

1 Jun 2026

PONE-D-25-52112R1

Public parks utilization and citizen satisfaction in Bangkok Metropolitan: An integrated theoretical model for tropical urban health

PLOS One

Dear Dr. Chanthanet,

Thank you for submitting your manuscript to PLOS ONE. After careful consideration, we feel that it has merit but does not fully meet PLOS ONE’s publication criteria as it currently stands. Therefore, we invite you to submit a revised version of the manuscript that addresses the points raised during the review process.

Please submit your revised manuscript by Jul 16 2026 11:59PM. If you will need more time than this to complete your revisions, please reply to this message or contact the journal office at plosone@plos.org. When you're ready to submit your revision, log on to https://www.editorialmanager.com/pone/ and select the 'Submissions Needing Revision' folder to locate your manuscript file.

Please include the following items when submitting your revised manuscript:

- A letter that responds to each point raised by the academic editor and reviewer(s). You should upload this letter as a separate file labeled 'Response to Reviewers'.
- A marked-up copy of your manuscript that highlights changes made to the original version. You should upload this as a separate file labeled 'Revised Manuscript with Track Changes'.
- An unmarked version of your revised paper without tracked changes. You should upload this as a separate file labeled 'Manuscript'.

If you would like to make changes to your financial disclosure, please include your updated statement in your cover letter. Guidelines for resubmitting your figure files are available below the reviewer comments at the end of this letter.

If applicable, we recommend that you deposit your laboratory protocols in protocols.io to enhance the reproducibility of your results. Protocols.io assigns your protocol its own identifier (DOI) so that it can be cited independently in the future. For instructions see: https://journals.plos.org/plosone/s/submission-guidelines#loc-laboratory-protocols. Additionally, PLOS ONE offers an option for publishing peer-reviewed Lab Protocol articles, which describe protocols hosted on protocols.io. Read more information on sharing protocols at https://plos.org/protocols?utm_medium=editorial-email&utm_source=authorletters&utm_campaign=protocols.

As the corresponding author, your ORCID iD is verified in the submission system and will appear in the published article. PLOS supports the use of ORCID, and we encourage all coauthors to register for an ORCID iD and use it as well. Please encourage your coauthors to verify their ORCID iD within the submission system before final acceptance, as unverified ORCID iDs will not appear in the published article. *Only*  the individual author can complete the verification step; PLOS staff *cannot*  verify ORCID iDs on behalf of authors.

We look forward to receiving your revised manuscript.

Kind regards,

Weicong Li, P.hD

Academic Editor

PLOS One

Journal Requirements:

If the reviewer comments include a recommendation to cite specific previously published works, please review and evaluate these publications to determine whether they are relevant and should be cited. There is no requirement to cite these works unless the editor has indicated otherwise.

Please review your reference list to ensure that it is complete and correct. If you have cited papers that have been retracted, please include the rationale for doing so in the manuscript text, or remove these references and replace them with relevant current references. Any changes to the reference list should be mentioned in the rebuttal letter that accompanies your revised manuscript. If you need to cite a retracted article, indicate the article’s retracted status in the References list and also include a citation and full reference for the retraction notice.

Additional Editor Comments:

Please find my comment in reviewer 3.

[Note: HTML markup is below. Please do not edit.]

Reviewers' comments:

Reviewer's Responses to Questions

**Comments to the Author**

1. If the authors have adequately addressed your comments raised in a previous round of review and you feel that this manuscript is now acceptable for publication, you may indicate that here to bypass the “Comments to the Author” section, enter your conflict of interest statement in the “Confidential to Editor” section, and submit your "Accept" recommendation.

Reviewer #1: All comments have been addressed

Reviewer #2: (No Response)

Reviewer #3: All comments have been addressed

**********

2. Is the manuscript technically sound, and do the data support the conclusions?

The manuscript must describe a technically sound piece of scientific research with data that supports the conclusions. Experiments must have been conducted rigorously, with appropriate controls, replication, and sample sizes. The conclusions must be drawn appropriately based on the data presented.

Reviewer #1: Yes

Reviewer #2: Partly

Reviewer #3: Partly

**********

3. Has the statistical analysis been performed appropriately and rigorously?

Reviewer #1: Yes

Reviewer #2: Yes

Reviewer #3: N/A

**********

4. Have the authors made all data underlying the findings in their manuscript fully available?

The PLOS Data policy requires authors to make all data underlying the findings described in their manuscript fully available without restriction, with rare exception (please refer to the Data Availability Statement in the manuscript PDF file). The data should be provided as part of the manuscript or its supporting information, or deposited to a public repository. For example, in addition to summary statistics, the data points behind means, medians and variance measures should be available. If there are restrictions on publicly sharing data—e.g. participant privacy or use of data from a third party—those must be specified.

Reviewer #1: Yes

Reviewer #2: Yes

Reviewer #3: No

**********

5. Is the manuscript presented in an intelligible fashion and written in standard English?

PLOS ONE does not copyedit accepted manuscripts, so the language in submitted articles must be clear, correct, and unambiguous. Any typographical or grammatical errors should be corrected at revision, so please note any specific errors here.

Reviewer #1: Yes

Reviewer #2: Yes

Reviewer #3: Yes

**********

6. Review Comments to the Author

Please use the space provided to explain your answers to the questions above. You may also include additional comments for the author, including concerns about dual publication, research ethics, or publication ethics. (Please upload your review as an attachment if it exceeds 20,000 characters)

Reviewer #1: This is a strong revision. The author has clearly taken the round-one comments seriously and worked through them carefully, one by one. The numbers that didn't add up before now do — the survey response rate is reported honestly (1,476 approached, 1,200 enrolled, 81.3%), and the previously missing diagnostics are all in place: VIF values, Harman's single-factor test, cluster-robust standard errors. Fig 1 is a real conceptual diagram now, not just a list of three theories sitting next to each other, and the empirical β coefficients embedded in it tie the framework to the actual results. The Bangkok evidence base, which was almost entirely absent before, is now properly engaged through five locally relevant studies, and the equity analysis — both socioeconomic and gendered — has become a genuine analytical thread running through the paper rather than a footnote. The causal claims have been dialed back to associational language throughout, the data availability statement now points to an institutional contact, and the Introduction and Conclusion have been restructured into flowing prose as Reviewer 3 asked. The response letter is detailed and point-by-point, which makes the revisions easy to verify.

Three things still need sorting before the paper is ready. The first is a real puzzle: the methods describe a research team — "each researcher," "two researchers independently coded 20% of transcripts (κ = 0.83)," "the research team developed integrated conclusions" — but the byline lists only one author. This needs to be clarified. If a research assistant did the second-coder work under the author's supervision, that should be stated plainly. If there was genuine analytical collaboration, the authorship statement needs to reflect that. As written, the two descriptions don't line up. The second concern is the Usage Patterns / Continued Usage Intention overlap. The two variables correlate at r = 0.708, which is high enough that readers will reasonably wonder whether the regression model is partly capturing the same construct twice. The author acknowledges this in the Limitations and gives a reasonable defense (current behavior vs. future intention; VIF = 1.89; distinct test-retest ICCs), but a short supplementary analysis — just showing what happens to the Usage Intention model when Usage Patterns is dropped — would settle the matter much more convincingly than argument alone. The third is the specific design parameters in the Discussion: 40% shade coverage, 20 lux pathway lighting, cooling stations every 150 m, water features of at least 500 m². These read as if they came out of the study, but the study didn't actually measure shade percentages, illuminance, or cooling effects. They appear to be engineering targets drawn from external tropical-design literature, which is fine — but they should be framed that way, not presented as findings. A small wording change ("Drawing on tropical urban design literature, the following parameters illustrate the magnitude of intervention implied by these findings…") would fix this.

A few smaller things are also worth a final pass. The R² benchmark of 40–55% for environmental psychology is still attributed to Cohen (1992), which is a general statistics source rather than a discipline-specific norm — either a better citation or softer framing ("typical R² values in behavioral cross-sectional research") would be more defensible. Reference [29] (Lai et al., 2009) is a disease-mapping text being used to support claims about tropical climate constraints on outdoor activity; Phosri et al. or Marks and Connell, both already in the manuscript, would fit better. The response letter has a few residual "ymy" typos from editing. And the abstract's framing of the R² as "substantially exceeding typical environmental psychology research (40–55%)" reads as a claim of model superiority, when in fact the higher R² is partly explained by self-report common-method effects and the predictor–outcome proximity issue — both of which the manuscript acknowledges elsewhere. A more neutral phrase would sit better with the paper's own honest acknowledgments.

Reviewer #2: The author provided some excellent additional data analysis and a deeper discussion of the theoretical framework linked to results. I believe these revisions have strengthened the paper and I only have a few very minor follow-up observations.

1. P. 43 of the revised manuscript, in relations to the AQI for BKK - it would be better to use the international year rather than the Thai year.

2. Figure 1 - does not appear in my pdf file.

3. The survey participant characteristics are interesting. I suspect that the salaries and education level (40% with a bachelors degree) are high even for the BMA, and certainly higher than the BMR or many other provinces in Thailand. What are the implications for your survey results, given these demographics? I do realized this is partially addressed in Study Limitations, but could be expanded upon.

4. For the multiple regressions, probably some of the independent variables (particularly the demographic variables) are significantly correlated. If this is the case, what are the implications for your results?

Reviewer #3: Some of the subjective statements in the “Results and Conclusions” section of the manuscript go beyond what is supported by the current evidence; please revise these statements.Some of the subjective statements in the “Results and Conclusions” section of the manuscript go beyond what is supported by the current evidence; please revise these statements.

**********

7. PLOS authors have the option to publish the peer review history of their article (what does this mean?). If published, this will include your full peer review and any attached files.

If you choose “no”, your identity will remain anonymous but your review may still be made public.

**Do you want your identity to be public for this peer review?**  For information about this choice, including consent withdrawal, please see our Privacy Policy.

Reviewer #1: No

Reviewer #2: No

Reviewer #3: No

**********

[NOTE: If reviewer comments were submitted as an attachment file, they will be attached to this email and accessible via the submission site. Please log into your account, locate the manuscript record, and check for the action link "View Attachments". If this link does not appear, there are no attachment files.]

To ensure your figures meet our technical requirements, please review our figure guidelines: https://journals.plos.org/plosone/s/figures

You may also use PLOS’s free figure tool, NAAS, to help you prepare publication quality figures: https://journals.plos.org/plosone/s/figures#loc-tools-for-figure-preparation.

NAAS will assess whether your figures meet our technical requirements by comparing each figure against our figure specifications.

# Author comment: Author response to Decision Letter 2

**DOI:** 10.1371/journal.pone.0354172.r004

5 Jun 2026

Dear Dr. Li,

Thank you for the opportunity to revise and resubmit my manuscript, "Public parks

utilization and citizen satisfaction in Bangkok Metropolitan: An integrated theoretical

model for tropical urban health" (PONE-D-25-52112R1).

I have carefully addressed all comments from the Academic Editor and the three reviewers.

A detailed, point-by-point response is provided in the "Response to Reviewers" file, and

all changes are marked in the "Revised Manuscript with Track Changes."

Key revisions in this round include:

- Clarified that this is a single-author study: trained research assistants contributed to

data collection and supervised second-coding only. The Methods wording was revised

accordingly and an Acknowledgments section was added (Reviewer #1).

- Added a supplementary sensitivity analysis (S1 Table) showing that the Continued Usage

Intention model is robust when Usage Patterns is removed, with quality remaining the

dominant predictor (Reviewer #1).

- Reframed the tropical design parameters (shade coverage, lighting, cooling stations,

water features) as illustrative targets drawn from the design literature, not as findings

of this study (Reviewer #1).

- Softened the framing of the model's R2 in the Abstract, Discussion, and Conclusions,

acknowledging common-method effects rather than implying model superiority (Reviewer #1).

- Corrected citations (the climate-constraint claim now cites Phosri et al. and Ng et al.;

an author-year citation was corrected to its numbered form) and replaced the Thai calendar

year with 2024 (Reviewers #1 and #2).

- Supplied Figure 1 as a separate file (Fig1.tif) per PLOS figure requirements, with the

caption retained in the manuscript text immediately after its first citation (Reviewer #2).

- Expanded the Limitations on sample representativeness (higher income and education) and on

inter-correlation among the demographic predictors (Reviewer #2).

- Tempered interpretive statements in the Results and Conclusions so they remain within what

the cross-sectional evidence supports (Reviewer #3 and the Academic Editor).

My financial disclosure statement is unchanged.

I believe these revisions have improved the clarity, rigor, and contribution of the

manuscript. Thank you for your time and consideration; I look forward to your feedback.

Sincerely,

Wichian Chanthanet

Faculty of Public Administration, Krirk University

wichian.cha@krirk.ac.th

Attachment Submitted filename: Response_to_Reviewers_Corrected_Clean.pdf

# Peer review: Decision Letter 2

**DOI:** 10.1371/journal.pone.0354172.r005

**Authors:** Weicong Li

5 Jul 2026

Public parks utilization and citizen satisfaction in Bangkok Metropolitan: An integrated theoretical model for tropical urban health

PONE-D-25-52112R2

Dear Dr. Chanthanet,

We’re pleased to inform you that your manuscript has been judged scientifically suitable for publication and will be formally accepted for publication once it meets all outstanding technical requirements.

Within one week, you’ll receive an e-mail detailing the required amendments. When these have been addressed, you’ll receive a formal acceptance letter and your manuscript will be scheduled for publication.

An invoice will be generated when your article is formally accepted. Please note, if your institution has a publishing partnership with PLOS and your article meets the relevant criteria, all or part of your publication costs will be covered. Please make sure your user information is up-to-date by logging into Editorial Manager at Editorial Manager® and clicking the ‘Update My Information' link at the top of the page. For questions related to billing, please contact billing support.

If your institution or institutions have a press office, please notify them about your upcoming paper to help maximize its impact. If they’ll be preparing press materials, please inform our press team as soon as possible -- no later than 48 hours after receiving the formal acceptance. Your manuscript will remain under strict press embargo until 2 pm Eastern Time on the date of publication. For more information, please contact onepress@plos.org.

Kind regards,

Weicong Li, P.hD

Academic Editor

PLOS One

Additional Editor Comments (optional):

-

Reviewers' comments:

Reviewer's Responses to Questions

**Comments to the Author**

1. If the authors have adequately addressed your comments raised in a previous round of review and you feel that this manuscript is now acceptable for publication, you may indicate that here to bypass the “Comments to the Author” section, enter your conflict of interest statement in the “Confidential to Editor” section, and submit your "Accept" recommendation.

Reviewer #1: All comments have been addressed

Reviewer #2: All comments have been addressed

**********

2. Is the manuscript technically sound, and do the data support the conclusions?

The manuscript must describe a technically sound piece of scientific research with data that supports the conclusions. Experiments must have been conducted rigorously, with appropriate controls, replication, and sample sizes. The conclusions must be drawn appropriately based on the data presented.

Reviewer #1: Yes

Reviewer #2: Yes

**********

3. Has the statistical analysis been performed appropriately and rigorously?

Reviewer #1: Yes

Reviewer #2: Yes

**********

4. Have the authors made all data underlying the findings in their manuscript fully available?

The PLOS Data policy requires authors to make all data underlying the findings described in their manuscript fully available without restriction, with rare exception (please refer to the Data Availability Statement in the manuscript PDF file). The data should be provided as part of the manuscript or its supporting information, or deposited to a public repository. For example, in addition to summary statistics, the data points behind means, medians and variance measures should be available. If there are restrictions on publicly sharing data—e.g. participant privacy or use of data from a third party—those must be specified.

Reviewer #1: Yes

Reviewer #2: Yes

**********

5. Is the manuscript presented in an intelligible fashion and written in standard English?

PLOS ONE does not copyedit accepted manuscripts, so the language in submitted articles must be clear, correct, and unambiguous. Any typographical or grammatical errors should be corrected at revision, so please note any specific errors here.

Reviewer #1: Yes

Reviewer #2: Yes

**********

6. Review Comments to the Author

Please use the space provided to explain your answers to the questions above. You may also include additional comments for the author, including concerns about dual publication, research ethics, or publication ethics. (Please upload your review as an attachment if it exceeds 20,000 characters)

Reviewer #1: (No Response)

Reviewer #2: The paper is clear, thorough, and should make a valuable contribution to the literature. The author has adequately addressed all of my comments and concerns. The only small suggest I would make is that the formatting of Figure 1 should ensure that all words are contained within their box boundaries.

Nicely done paper!

**********

7. PLOS authors have the option to publish the peer review history of their article (what does this mean?). If published, this will include your full peer review and any attached files.

If you choose “no”, your identity will remain anonymous but your review may still be made public.

**Do you want your identity to be public for this peer review?**  For information about this choice, including consent withdrawal, please see our Privacy Policy.

Reviewer #1: No

Reviewer #2: No

**********

# Editor report: Acceptance letter

**DOI:** 10.1371/journal.pone.0354172.r006

**Authors:** Weicong Li

PONE-D-25-52112R2

PLOS One

Dear Dr. Chanthanet,

I'm pleased to inform you that your manuscript has been deemed suitable for publication in PLOS One. Congratulations! Your manuscript is now being handed over to our production team.

At this stage, our production department will prepare your paper for publication. This includes ensuring the following:

* All references, tables, and figures are properly cited

* All relevant supporting information is included in the manuscript submission,

* There are no issues that prevent the paper from being properly typeset

You will receive further instructions from the production team, including instructions on how to review your proof when it is ready. Please keep in mind that we are working through a large volume of accepted articles, so please give us a few days to review your paper and let you know the next and final steps.

Lastly, if your institution or institutions have a press office, please let them know about your upcoming paper now to help maximize its impact. If they'll be preparing press materials, please inform our press team within the next 48 hours. Your manuscript will remain under strict press embargo until 2 pm Eastern Time on the date of publication. For more information, please contact onepress@plos.org.

You will receive an invoice from PLOS for your publication fee after your manuscript has reached the completed accept phase. If you receive an email requesting payment before acceptance or for any other service, this may be a phishing scheme. Learn how to identify phishing emails and protect your accounts at https://explore.plos.org/phishing.

If we can help with anything else, please email us at customercare@plos.org.

Thank you for submitting your work to PLOS ONE and supporting open access.

Kind regards,

PLOS ONE Editorial Office Staff

on behalf of

Dr. Weicong Li

Academic Editor

PLOS One
